# Multi-technique characterization of iron reduction by an Antarctic *Shewanella*: an analog system for putative Martian biosignature identification

**DOI:** 10.1128/aem.02528-24

**Published:** 2025-07-10

**Authors:** Jacob M. C. Shaffer, Elizabeth C. Sklute, Robert M. Samples, Lesley-Ann Giddings, Abigail Jarratt, Katherine Mateos, M. Darby Dyar, Peter A. Lee, Kenneth J. T. Livi, Jill A. Mikucki

**Affiliations:** 1 Department of Microbiology, University of Tennessee - Knoxville 189504, Knoxville, Tennessee, USA; 2 Los Alamos National Laboratory 5112, Los Alamos, New Mexico, USA; 3 Planetary Science Institute 53469, Tucson, Arizona, USA; 4 Department of Chemistry, Smith College 6089, Northampton, Massachusetts, USA; 5 Grice Marine Laboratory, College of Charleston 539249 , Charleston, South Carolina, USA; 6 Department of Astronomy, Mount Holyoke College 7397, South Hadley, Massachusetts, USA; 7 Hollings Marine Laboratory, College of Charleston 2343, Charleston, South Carolina, USA; 8 Department of Materials Science and Engineering, Johns Hopkins University 310945, Baltimore, Maryland, USA; Colorado School of Mines, Golden, Colorado, USA

**Keywords:** biosignatures, microbial iron reduction, Antarctic *Shewanella*, early Mars, volatile organic compounds, transcriptomics

## Abstract

Microbes from terrestrial extreme environments enable testing of biosignature production in conditions relevant to astrobiological targets. Mars, which was likely more conducive to life during early warmer and wetter epochs, has inspired missions that search for signs of early life in the surficial rock record, including mineral or organic biosignatures. Microbial iron reduction is a common and ancient metabolism that may have also operated on other rocky celestial bodies. To investigate biosignature production during iron reduction, a *Shewanella* sp. (strain BF02_Schw) isolated from a subglacial discharge known as Blood Falls, Antarctica, was incubated with the electron acceptor ferrihydrite (Fh). Biosignatures associated with Fh reduction were identified using a suite of techniques currently utilized or proposed for Mars missions, including X-ray diffraction and infrared, Mössbauer, and Raman spectroscopy. The biotic origin of features was validated by transcriptional changes observed between treatments with and without Fh and comparison to killed controls. In live treatments, Fh was reduced to magnetite and goethite, both detected in Martian lacustrine basins. Several soluble and volatile metabolites were also detected, including riboflavin and dimethyl sulfide (DMS), which could be astrobiological indicators of active microbial processes. While none of the identified biosignatures individually would serve as definitive proof of life (past or present), detecting concomitant features associated with known terrestrial biotic processes would provide compelling rationale for more targeted life detection missions. Terrestrial extremophiles can support the exploration of astrobiologically relevant microbial processes, validation of life detection instrumentation, and potentially the discovery of new biomarkers.

**IMPORTANCE:**

Culture-based experiments with terrestrial extremophiles can elucidate biosignatures that may be analogous to those produced under extraterrestrial conditions, and thus inform sampling and technology strategies for future missions. Here, we demonstrate the production of several biosignatures under iron-reducing conditions by *Shewanella* sp. BF02_Schw, originally isolated from an Antarctic analog feature. These biosignatures could be detectable using flight-ready instrumentation. Growth experiments with terrestrial extremophiles can identify biosignatures measurable by current methodologies and inform the development and optimization of techniques for detecting extant or extinct life on other worlds.

## INTRODUCTION

Any life that emerged on Mars likely occurred during warmer, wetter climates of its early history (i.e., the Noachian or Hesperian eons) (1). Decades of Martian orbital and surface missions revealed evidence of historic liquid water at the surface (1–3) associated with possible redox gradients, which would be available for biological exploitation (4). While habitability on Mars may have been transient (5), it is possible life emerged independently of Earth’s life, or that life emerged first on Mars and then seeded early Earth in a panspermia event (1) or vice versa. However, the Martian surface today is cold, dry, and likely uninhabitable with the possible exception of localized Special Regions (6).

Recent missions to Mars have sought to identify evidence of ancient habitability (7). Because water is crucial to life as we know it, both the *Curiosity* and *Perseverance* rovers landed at sites with evidence of ancient fluvial and lacustrine settings (8, 9). On Earth, depositional environments also supported the oldest generally accepted microbial fossils, stromatolites, layered sedimentary features originating from interactions between sedimentary particles and biofilm communities (10, 11). Such environments may also lead to the preservation of diverse biosignatures, including microbially produced secondary minerals, micro- or macro-scale textures, and preserved structural components of biomolecules such as lipids (12). While identifying a singular biosignature in a potentially habitable region would be intriguing, concomitant biosignatures would be significantly more compelling.

Early Earth studies can inform life detection during Noachian or Hesperian Mars. Evidence for life in Earth’s Precambrian oceans exists largely as chemical (e.g., complex organics, isotopic composition) or morphological biosignatures within sedimentary basins (13, 14). Accurate interpretation of these records is challenging due to the rare and incomplete nature of fossilization, abiotic mineral alterations, and the inherent ambiguity of mineral biosignatures, which often have both biotic and abiotic routes of formation. Thus, claims of evidence of early Earth life rely on multiple observations to rule out abiotic formation, favoring biogenesis (14). These lines of evidence take many forms, including both macro- and microscale morphological characteristics (10), isotopic composition (15), the presence of microfossils (16), and secondary mineral abundance and composition (17). Similar concerns apply to the study of Mars (18). While regional morphology, possible microfossils, and mineral structures have been proposed as potential evidence for Martian life, they remain controversial (e.g., 19, 20). Methods that can better elucidate complex biosignatures or more clearly confirm their biogenic origin would strengthen claims of both ancient and extant life, especially those associated with the production of biogenic mineral biosignatures.

Martian planetary science missions utilize diverse payloads of instruments to assess present and past habitability. The earliest attempts at measuring life *in situ* were conducted by the *Viking* missions, which included experiments designed to detect active metabolic processes in Martian soils through respiration or pyrolytic release of labeled and unlabeled compounds (21). Of the three deployed biological experiments, only the Labeled Release experiments, in which soils were enriched with ^14^C-labeled organics and monitored for radioactive gas production, presented a possible positive result (i.e., gas evolution following injection that was quenched by sample heating; 22, 23), though interpretation of these results remains highly controversial (24, 25). Since the *Viking* missions, surface landers and rovers have focused on morphological and geochemical characterization, with an emphasis on determining whether Mars was previously habitable. To this end, several instruments have been deployed to describe mineral and organic features, including possible biosignatures such as chemistry or morphology indicative of ancient metabolic processes. Visible and near-infrared (VNIR) spectroscopy has been used both by rovers (26, 27) and orbiters, such as the Compact Reconnaissance Imaging Spectrometer for Mars (CRISM) on the Mars Reconnaissance Orbiter (MRO; 28), and the Observatoire pour la Minéralogie, l'Eau, les Glaces et l'Activité (OMEGA) instrument on *Mars Express* (29). Mid-far infrared spectroscopy has similarly been employed on the Mars Exploration (MER) rovers (30), on Mars Global Surveyor (31), and on Mars Odyssey (32). The MERs were also equipped with Mössbauer spectrometers specifically for the identification of Fe-bearing minerals (33). The Mars Science Laboratory (MSL) rover *Curiosity* features both X-ray diffraction (XRD) and limited fluorescence (XRF) capabilities as a part of the ChemMin instrument (34), and XRF was also deployed on the Mars 2020 PIXL (Planetary Instrument for X-ray Lithochemistry) instrument (35). Both ChemMin and PIXL are designed for the identification of surface mineral elemental chemistry. *Curiosity* also features the SHERLOC (Scanning Habitable Environments with Raman & Luminescence for Organics and Chemicals) Raman and fluorescence spectrometer (36), in addition to the SAM (Sample Analysis at Mars) instrument suite for characterization of organic components and isotopic states of surface materials, including a gas chromatograph, laser spectrometer, and quadrupole mass spectrometer (37). Recently, SAM detected pmol concentrations of alkanes possibly originating from long-chain carboxylic acids, though whether the molecules were biotic or abiotic in origin remains unknown (38). While no recently deployed instruments alone are likely to detect biosignatures that preclude an abiotic origin, when utilized in conjunction, the possibility remains that concomitant chemical complexities and morphological features may provide more compelling evidence of ancient life.

Similar to how terrestrial analog features are utilized to test astrobiological instrumentation and hypotheses, terrestrial microorganisms isolated from analog environments are beneficial for exploring possible adaptations and metabolic processes associated with early Earth and Mars. *Shewanella* spp. have long been utilized as model organisms for studying bacterial Fe transformation (39). Under anaerobic conditions, *Shewanella* spp. can utilize a wide variety of terminal electron acceptors, including a number of Fe(III) species like amorphous ferrihydrite (Fh; 39). Dissimilatory iron reduction (DIR), which mediates the reduction of Fe(III) to Fe(II) via external electron transfer (40), is believed to be an ancient metabolic pathway on Earth (41). Because Fh and other Fe(III) (hydr)oxides are unable to diffuse or be transported through cellular membranes, DIR by *Shewanella* spp. relies on membrane-bound redox machinery and soluble electron carriers (42). Electrons are transferred from the inner membrane electron transport chain to insoluble Fe(III) oxides through the Mtr complex, a series of heme-bearing proteins localized in the inner and outer membranes. Electrons may also be passed to soluble electron shuttles, including flavins, that discharge electrons to external sources (43). *Shewanella* spp. have also served as model organisms for astrobiology; Malas et al. (44) investigated transcriptional changes in *Shewanella oneidensis* MR-1 when exposed to Titan-relevant pressure to understand survivability and possible adaptations for living in sub-ice oceans. *S. oneidensis* MR-1 was also shown to reduce Fe(III) present in a simulated Martian regolith (45), supporting the hypothesis that DIR is a plausible metabolic strategy for Martian life (46).

To further investigate the production of mineral and chemical biosignatures during bacterial DIR, we utilized a psychro- and halotolerant *Shewanella* strain (BF02_Schw), isolated from an Fe-rich subglacial brine feature in Antarctica known as Blood Falls (47, 48). Biogenic mineral transformations and the production of associated soluble and volatile organic compounds (VOCs) were measured using mission-relevant techniques (49). To link observed “biosignatures” to biotic processes, the transcriptome of BF02_Schw grown with and without Fh was sequenced to identify genes associated with the production of observed mineral and chemical features.

## RESULTS

### Growth of *Shewanella* sp. BF02_Schw

Growth of BF02_Schw was observed in media containing Fh + lactate + thiosulfate (FLT), lactate + thiosulfate (LT), and Fh + lactate (FL; Table 1). Growth in microcosm experiments was confirmed by observing an increase in viable counts (i.e., colony-forming units or CFUs) over time and accumulation of reduced iron Fe(II) in incubations containing Fh. While these measurements indicate growth, they may undercount viable populations still attached to mineral particles after vortexing. At the termination of experiments, all Live treatments (Table 1) displayed an increase in biomass over time (Fig. S1). At the time of inoculation, Live BF02_Schw microcosms contained an average of 10^6^ CFU mL^−1^ across all experiments. Both 2- and 8-week Live incubations contained ~10^8^ CFU mL^−1^. Viable cells were not observed in the No Cell and Dead controls.

**TABLE 1:** Summary of utilized treatments^*a*^

Treatment	Components
FLT	Minimal medium + Fh + lactate + thiosulfate
LT	Minimal medium + lactate + thiosulfate
FL	Minimal medium + Fh + lactate (used for mineralogy only)

^*a*^ Fh, ferrihydrite.

Live FLT treatments showed a significant increase in Fe(II) following both 2- and 8-week incubations (Fig. S2 and S3). A visible transformation from reddish-brown to black was observed in all Live FLT incubations after 2 weeks (Fig. S2a). Insoluble black mineral products were magnetic (Fig. S2b). A transient increase in both soluble Fe(II) and Fe(III) was observed in Live treatments after 2 weeks, but not 8 weeks (Fig. S3a). Live incubations with thiosulfate showed increased Fe reductions compared to other incubations (i.e., FL; Fig. S3b).

### Mineral transformations

**Fig 1:**
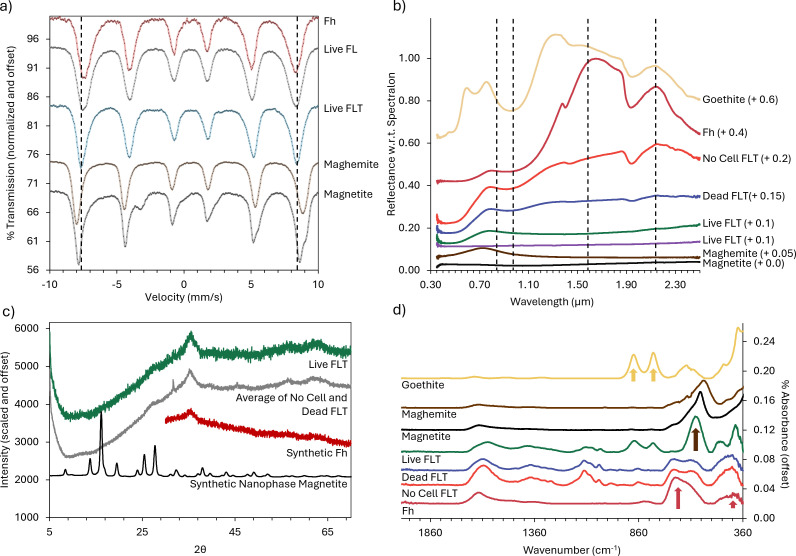
Summary of mineralogical changes following 2 weeks of incubation. (**a**) Mössbauer spectra for all Live treatments. Vertical lines highlight the shift in peaks consistent with the presence of Fe^2+^. (**b**) VNIR spectra for FLT treatments. Two Live FLT treatments are shown due to slight differences in absorption. (**c**) XRD spectra for Live cells in FLT medium compared to averaged Dead and No Cell controls. Observed features are largely consistent with Fh. (**d**) Fourier transform infrared (FTIR) spectra of FLT treatments. Standard peaks consistent with observed features in treatments are highlighted with arrows. Utilized media are described in Table 1.

Coordinated diffraction and spectroscopic techniques were used to determine changes in mineralogy following incubation. Because each technique employs a different energy of impending radiation to query the sample, each discerns a different type of information (Fig. 1). As preliminary evidence suggested that thiosulfate increased Fh reduction by BF02_Schw (Fig. S3b), an additional Fh-containing treatment (FL) was utilized for mineralogy experiments (Table 1).

#### X-ray diffraction

XRD data from all samples showed two broad peaks characteristic of Fh and did not provide clarity on mineral transformations (Fig. 1c; Fig. S4). Because XRD is a bulk technique and the bio-reduced transformations are likely surface phenomena, this result indicates that, volumetrically, changes were minor.

#### Fourier transform infrared spectroscopy

Fourier transform infrared (FTIR) results (Fig. 1d) showed that both No Cell and Dead control spectra were similar to reference Fh but contained organic signatures resulting from the medium. Live FLT cultures showed significant transformation into a combination of goethite and magnetite after 2 weeks of incubation, with a larger relative proportion of magnetite after 8 weeks (Fig. S5 and S6). For these products, the main magnetite peak was shifted to longer wavenumbers with respect to pure, synthetic magnetite, and an additional peak at 475 cm^−1^ not directly attributable to either (hydr)oxide was apparent. In Live FL cultures, magnetite and goethite were only present following 8 weeks of incubation with a primary absorption at 560 cm^−1^, consistent with the position and appearance of synthetic magnetite.

#### VNIR spectroscopy

VNIR spectra of No Cell FLT treatments showed minor spectral alteration of starting Fh resulting from reaction with media components during incubation (Fig. 1b; Fig. S7). Products from Live FL treatments showed the least spectral change, with only slight muting of overall spectral contrast, whereas products from Live FLT treatments were significantly more muted. For all conditions, peak positions remained consistent except for the absorption at ~1.403 µm in pure Fh spectra, which shifted to ~1.444 µm in all incubated samples. Products from dead incubations lost significant spectral contrast, flattening at wavelengths > ~1 µm. All Fh-like absorptions near 0.92 µm, 1.444 µm, and 1.937 µm, along with the VIS maximum at 0.791 µm, remained visible. The loss of spectral contrast would make the ~0.92 µm absorption appear to shift in continuum-removed spectra. All products from Live treatments are spectrally dark (<~0.1 reflectivity with respect to Spectralon) with a loss of Fh-like features between the VIS maximum and a small NIR maximum ~2.142 µm, which is replaced with either a long flat (FL) or convex (FLT) appearance likely due to multiple overlapping absorptions, including those for Fe^2+^. One replicate of the FLT Live experiment was absorbed throughout the VNIR spectrum and was consistent with magnetite.

#### Raman spectroscopy

Bulk Raman spectra were overwhelmed by fluorescence from the Fe-bearing starting material (Fig. S8). Spectra of the experimental samples were compared to Raman spectra of sulfates, sulfides, carbonates, and (hydr)oxides (Fig. S8a), but no phase was immediately identifiable as a reaction product. Prominent features at ca. 428 and 492 cm^−1^ appeared in all samples except for a single analysis of the Live FL treatment (Fig. S8b). These peaks cannot be assigned to a specific mineral phase and are assumed to be due to elements of the media. In the Live FL incubation, there was a peak at 336 cm^−1^ common to most sulfides, but could not be assigned to a specific phase. Sulfides with partial matches include greigite (peak at 350 cm^−1^), pyrite (doublet at 348 and 384 cm^−1^), and pyrrhotite (348 and 380 cm^−1^). An additional prominent peak in that sample at 524 cm^−1^ was not a match to any of the reference phases analyzed. Of the (hydr)oxides identified by other techniques used in this paper, none of the main features arising from hematite (26, 294, and 408 cm^−1^), akaganéite (307 and 387 cm^−1^), goethite (305 and 397 cm^−1^), magnetite (670 cm^−1^), maghemite (673 and 709 cm^−1^) and Fh (716 cm^−1^) were apparent.

#### Mössbauer spectroscopy

Mössbauer spectra provided specific details on the nature of the Fe transformation in Live treatments and confirmed the minimal transformation in Dead and No Cell treatments (Table S1 and S2). Evidence for Fe^2+^ was only seen in Live FLT treatments (Fig. 1a; Fig. S9).

#### Transmission electron microscopy

**Fig 2:**
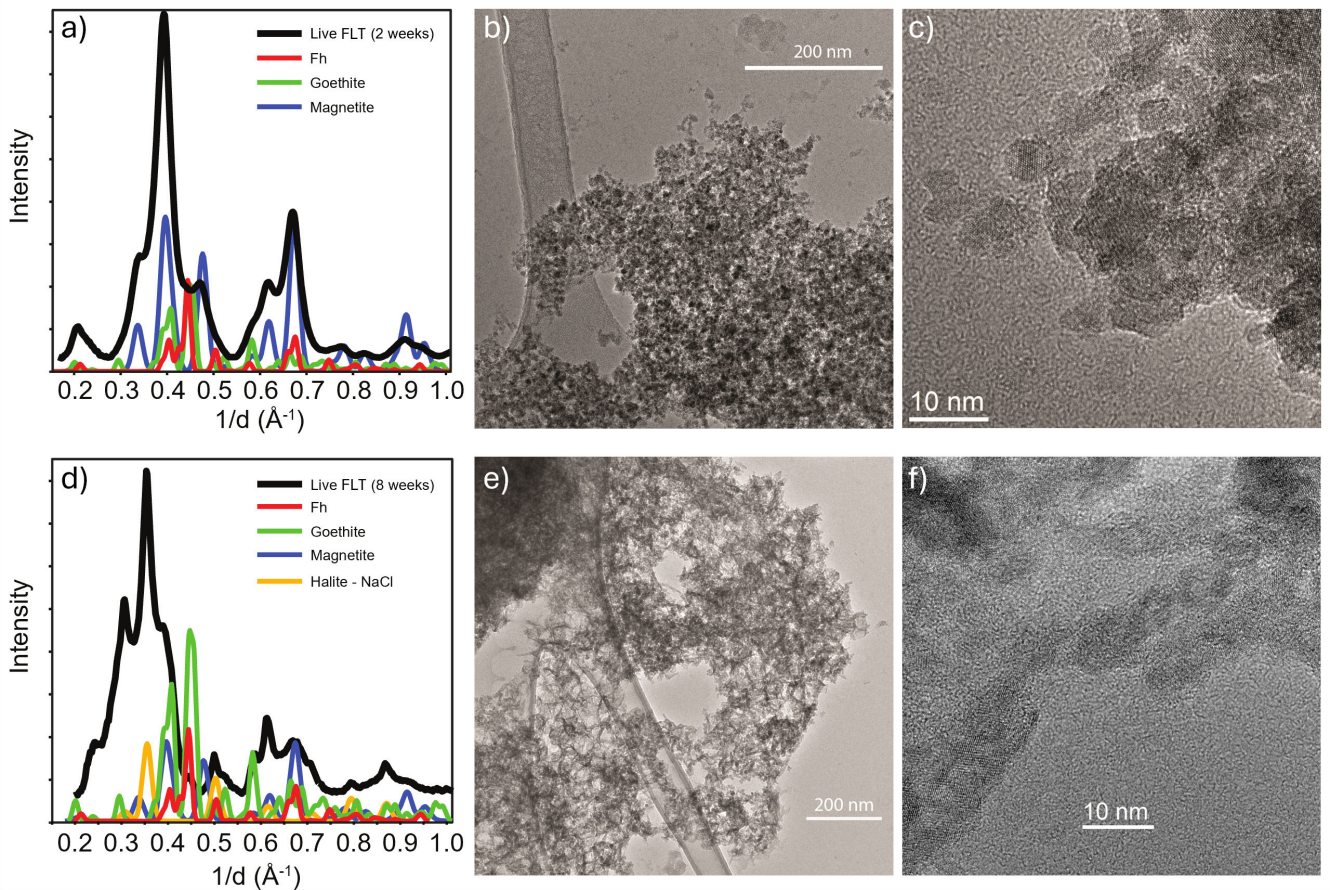
Identification of mineralogy of Live FLT treatments following 2 weeks (**a–c**) and 8 weeks of incubation (**d–f**) by TEM and SAED. (**a**) Two-week incubations feature peaks consistent with both magnetite and Fh, with minor peaks associated with goethite, as seen in both (**b**) TEM images and (**c**) SAED diffraction patterns. (**d**) Eight-week incubations show an increase in features consistent with goethite, in addition to the presence of magnetite and the appearance of peaks consistent with halite formation, which are consistent with both (**e**) TEM images and (**f**) SAED patterns.

Transmission electron microscopy (TEM) of both 2- and 8-week incubations showed evidence of Fe transformation (Fig. 2) and visible association between cells and Fe particles (Fig. S10). Signatures of Fh were present in all Live treatments. Although visually similar, selected area electron diffraction (SAED) ring diffraction of the Live FLT culture predominantly showed magnetite with some Fh after 2 weeks with the addition of goethite, seen as a shoulder at 0.23 1/Å, after 8 weeks (Fig. 2d). Moreover, there are subtle differences between treatments at 2 weeks. Fh, magnetite, and akaganéite were present in the FL sample (Fig. S11).

### Transcriptional changes

**Fig 3:**
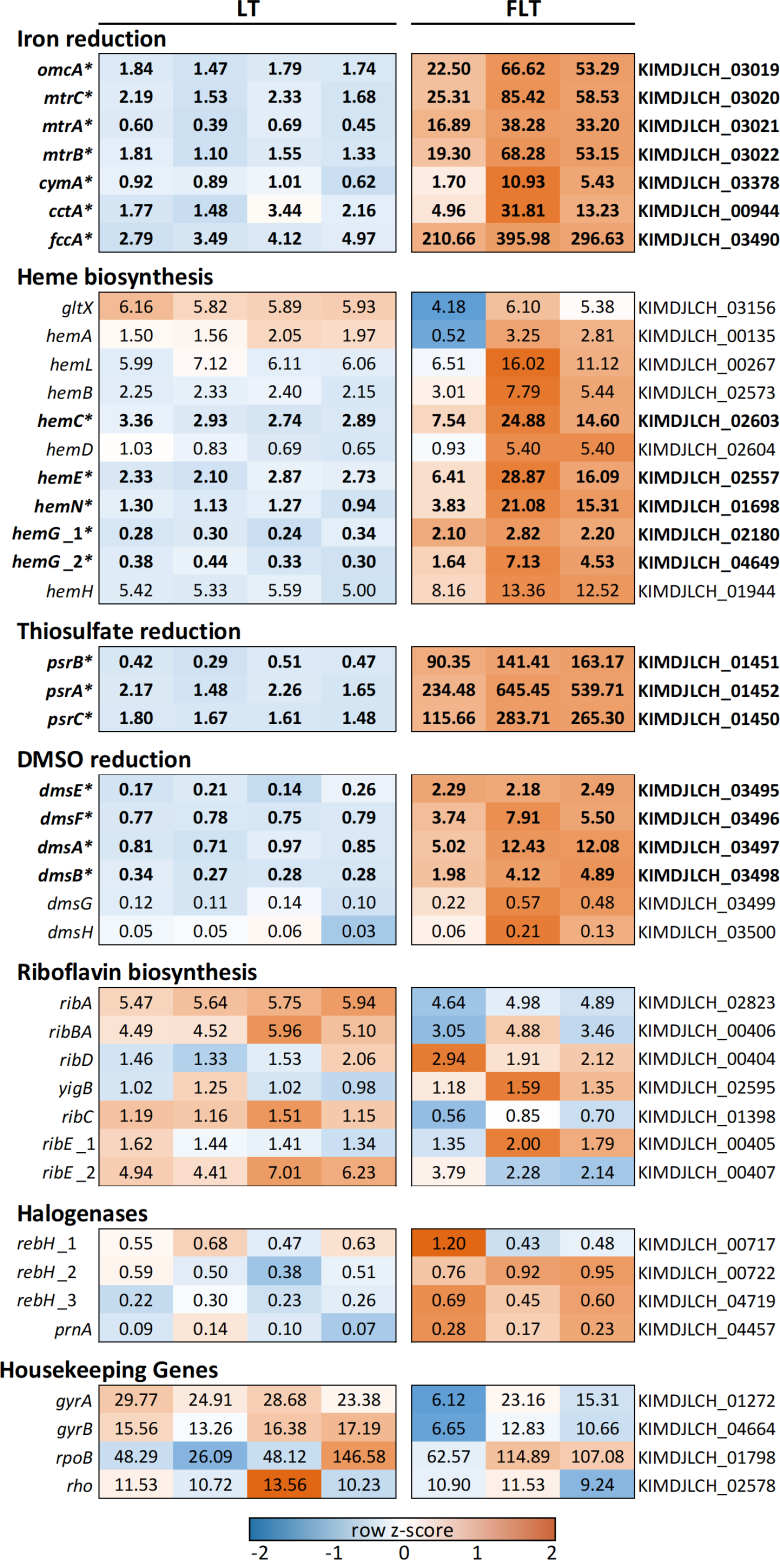
Abundance of transcripts of interest between FLT and LT treatments following 2 weeks of incubation. Transcript identities were determined based on both annotation by Prokka (51) and sequence alignment to characterized genes and are grouped by putative function. Numeric values for each sample represent normalized transcript abundance (median of ratios) per thousand transcripts. Shading represents the Z-score for each gene (standard deviations from the mean). Only three FLT replicates were sequenced due to low RNA quality. Marked transcripts (*) are significantly different between treatments.

RNA sequencing was utilized to validate the biogenicity of observed biosignatures. RNA integrity (RIN) numbers from 2-week incubated samples ranged from 8.1 to 9.0 in LT treatments and from 5.3 to 6.3 in FLT treatments. One FLT replicate was not sequenced due to low quality, highlighting the challenge of extraction from cultures containing 100 mM Fe. Despite multiple attempts, we were unable to extract RNA from 8-week FLT Live treatments of sufficient quality for sequencing and will only present results from 2-week treatments. Sequencing of 2-week Live treatments yielded 248,789,368 total reads (average of 33,696,840 reads for three FLT replicates and 36,924,712 reads for four LT replicates). An average of 79.45% of sequencing reads aligned with the published BF02_Schw genome. Read processing yielded an average of 18,627,177.99 normalized reads for LT treatments and 26,213,974.72 normalized reads for FLT treatments associated with 4,637 genes in the BF02_Schw genome, with 97.9% coverage. Complete transcription results are reported in Table S3. Of these, 460 genes had significantly different transcript abundance (as log_2_ fold change; LFC ≥1 or ≤ −1, *P*_adj_ ≤0.05), with 318 transcripts more abundant in FLT treatments (LFC ≥1) and 142 transcripts less abundant in FLT treatments (LFC ≤ −1). An additional 974 transcripts were considered non-significantly differentially abundant (LFC ≥1 or ≤ −1, *P*_adj_ >0.05). Complete transcription results are reported in Table S3. Differential abundance was validated by comparison of multiple *Shewanella* housekeeping genes, including *gyrA*, *gyrB*, *rpoB*, and *rho* (50), none of which were significantly different between treatments (Fig. 3; Table S4).

Of significantly different transcripts, 287 were annotated as non-hypothetical. Of the 202 transcripts with greater abundance, the most represented pathway categories were carbohydrate metabolism (38 genes), protein families: signaling and cellular processes (20), unclassified: metabolism (20), environmental information processing (18), and energy metabolism (16). Only 78 of the significantly less abundant transcripts were annotated, with the largest categories belonging to protein families: genetic information processing (13), genetic information processing (8), environmental information processing (7), amino acid metabolism (7), and unclassified: metabolism (7).

Transcripts associated with anaerobic Fh metabolism were significantly higher in FLT treatments (Fig. 3; Table S4). Genes for the canonical *Shewanella* metal reduction complex (*mtr*) were identified in the BF02_Schw genome via BLAST and included *omcA*, *mtrC*, *mtrA*, *mtrB*, *cymA, fccA, and cctA* (42, 52). Transcripts for the second half of the heme biosynthesis pathway (identified via KEGG map M00121) were significantly higher in FLT incubations, with the exception of *hemB*, *hemD*, and *hemH*, which were higher but not significant (Fig. 3; Fig. S12a; Table S4). Significantly more abundant *hem* included *hemC*, *hemE*, *hemN*, *hemG*_1, and *hemG*_2.

Genes associated with *psr* thiosulfate reductase had some of the highest LFC observed in FLT treatments (Fig. 3; Table S4) and included *psrA*, *psrB*, and *psrC* (53). Transcripts associated with dimethyl sulfoxide (DMSO) reduction were more highly transcribed. Significantly more abundant genes included *dmsE*, *dmsF*, *dmsA*, and *dmsB* (54). Transcripts for *dmsG* and *dmsH* were more abundant in +Fh treatments, though not significantly.

Other genes of interest were transcribed but were not significantly different between treatments, including riboflavin biosynthesis (*ribA*, *ribBA*, *ribD*, *yigB*, *ribC*, *ribE*_1, and *ribE*_2; Fig. 3; Fig. S12b; Table S4). In addition, four putative flavin-dependent halogenases, *rebH_1*, *rebH_2*, *rebH_3*, and *prnA*, were identified within transcripts.

### Soluble metabolites

Soluble metabolites were detected via ultra-high purity liquid chromatography mass spectrometry (UHPLC-MS) in supernatant collected from 2-week Live LT treatments and both 2- and 8-week Live FLT treatments. Following peak alignment, 18,514 features were present amongst all samples, with 725 (3.92%) features passing all quality filters. Of these features, 306 were present in all treatments; LT 2-week and FLT 8-week treatments had 42 unique features each, and 41 features were present in only FLT treatments (2- and 8-week incubations). No features were unique to the 2-week FLT supernatant. Based on hierarchical clustering analysis of normalized abundance of molecular features, extraction and injection replicates grouped, and LT treatments formed a separate statistical clade from FLT treatments (Fig. S13).

**Fig 4:**
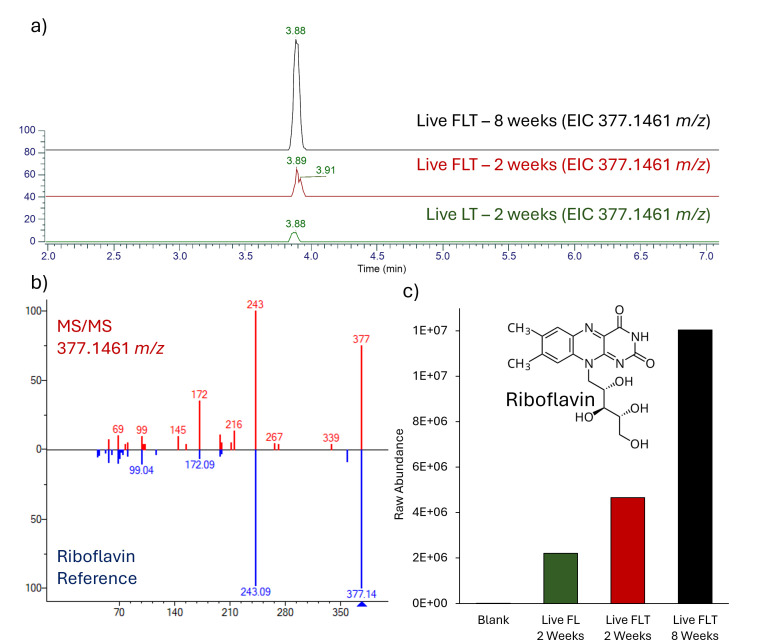
Differential abundance of riboflavin detected via UHPLC-MS. (**a**) Extracted ion chromatograms (EIC) for a feature with m/z 377.1461. (**b**) Mirrored MS/MS fragmentation patterns for predicted riboflavin feature mirrored with a known standard for riboflavin. (**c**) Raw abundance of putative riboflavin features across the examined supernatants. (inset) Molecular structure of riboflavin.

Riboflavin was identified within supernatant extracts via comparison to an authentic standard (Fig. 4). A putative riboflavin feature (*m/z* 377.1461; calculated for C_17_H_21_N_4_O_6_ [M + H]^+^, 377.1462) was detected in all microcosm extracts with a retention time of 3.88–3.89 (Fig. 4a). The MS/MS spectra of the peak were congruent with the fragmentation pattern obtained from the standard (Fig. 4b). This feature was present in greater abundance in FLT incubations compared to LT supernatant (Fig. 4c).

### Volatile metabolites

**Fig 5:**
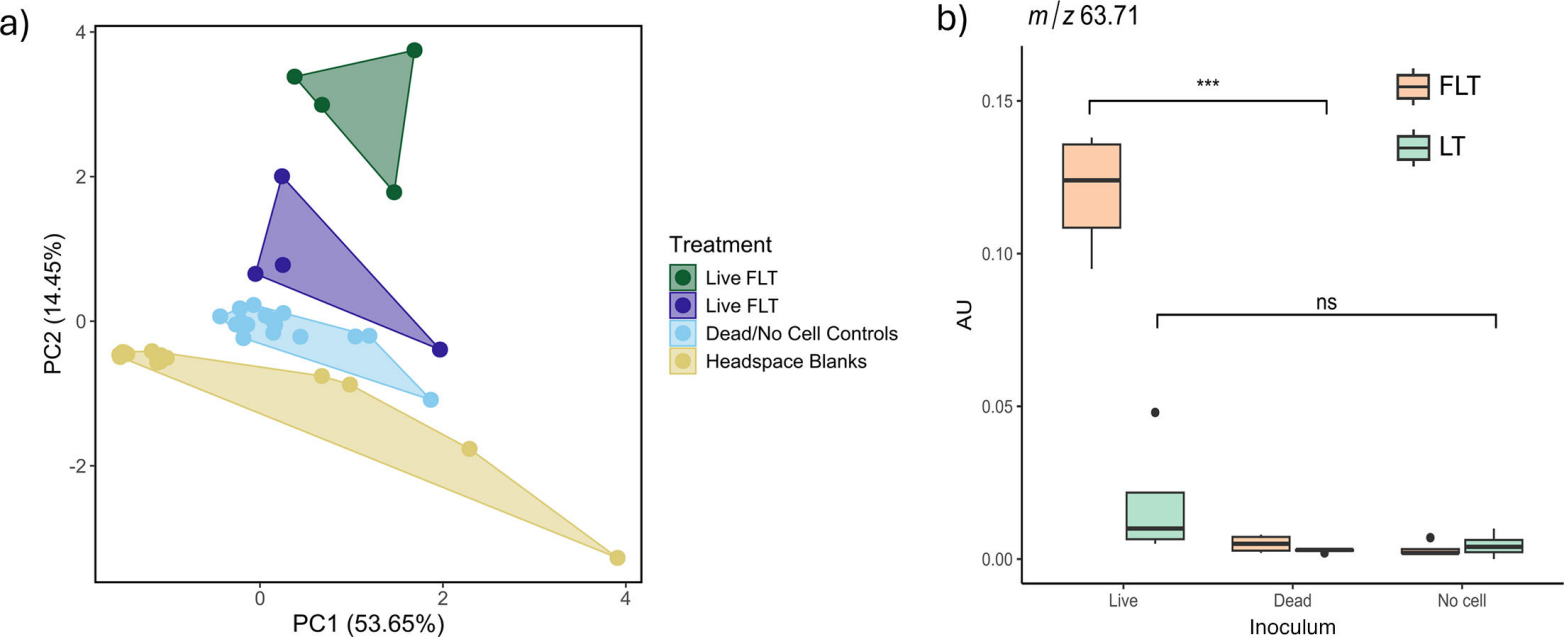
VOCs detected in microcosm headspace following 8 weeks of incubation. (**a**) PCA plot showing differences between treatments. Polygons represent the range of observed scores within a treatment group. (**b**) Differential abundance of feature with *m/z* 63.71, putatively identified as DMS. ns, not significant.

Throughout incubation, VOC profiles detected in microcosm headspace differed between treatments (Fig. 5a). In FLT and LT Live treatments, 18 features were significantly higher compared to Dead, No Cell, and N_2_ controls (Table S5; *P* < 0.001 for all). After 2 weeks, three features were more abundant in Live treatments compared to controls, and one feature was significantly more abundant in Live LT treatments. After 8 weeks, 10 features were significantly higher in both Live treatments compared to No Cell and Dead controls (*P* < 0.001 for all). Of these features, five were significantly higher in FLT compared to LT. An additional four features were significantly higher in Live + Fh only. Of all features, 38 were putatively identified (Fig. 5a; Table S5). One feature (*m/z* 63.71) was putatively identified as dimethyl sulfide (DMS), ethanethiol, or 1,2-ethanediol, and was significantly more abundant in +Fh Live headspace following 8 weeks (Fig. 5b). Identity of the feature as DMS was supported by comparison to a DMS standard.

Separate experiments showed that BF02_Schw cultures produce DMS from DMSO, where D_6_-DMS was detected via membrane inlet mass spectrometry (MIMS) following incubation with D_6_-DMSO (Fig. S14). However, the media for the microcosms presented here did not contain DMSO. The high concentration of iron oxides and thiosulfate found within FLT media likely generates oxidative stress, which may, in turn, drive non-canonical sulfur transformations that ultimately result in volatile thiol production; however, this theory would require additional investigation.

## DISCUSSION

### Biogenic Fe reduction

Microbe-mineral interactions through metabolism or adhesion to mineral surfaces can alter mineral surface texture, crystal structure, inclusion of organics, or generate distinct mineral species (55). For example, aqueous Fe(II) is produced during DIR and adsorbs to unreduced Fe(III), leading to the formation of secondary Fe minerals such as goethite, hematite, and magnetite (56). While secondary Fe minerals can form abiotically, biogenic Fe mineral phases can be identified by morphological and compositional features that persist in the rock record. Terrestrially, secondary Fe minerals, including hematite and magnetite, have been proposed as possible products of DIR in Archaean Banded Iron Formations (e.g., 17, 57–59). While an abiotic origin cannot be precluded (60), mineralogical features from Fe-metabolizing organisms in Earth’s Banded Iron Formations may help interpret features detected on Mars as possible signs of life. On Mars, Fe mineralogy and its implications for extant or extinct life have been the subject of multiple investigations. For example, the MSL ChemMin instrument identified stratified magnetite and hematite abundance within Gale Crater, supporting the presence of a redoxycline in the putative Hesperian lake (4, 61). While these minerals are hypothesized to exist due to abiotic processes, redox gradients are key to life on Earth, suggesting Gale Crater may have hosted a habitable lake (4, 62). Much of Martian mineralogy remains unresolved due to the abundance of X-ray amorphous non-crystalline materials that are difficult to identify with XRD and contain various Fe species, including Fh (4). Fh has been identified within Martian meteorites dated to the Amazonian (63) and possibly the Noachian (64), suggesting that Fh has been present on Mars at different points throughout its history. The timing of the formation of these meteorites suggests that oxidized Fe minerals were available as potential terminal electron acceptors on early Mars (46).

*Shewanella* spp. are widely utilized to study microbial DIR and thus can provide insight into mineral biosignature formation (39). A variety of Fe(II), Fe(III), and Fe(II, III) species have all been observed as products of Fh reduction by *Shewanella* spp (56, 65, 66), though the exact composition of mineral products is highly dependent on environmental context (67). In our BF02_Schw microcosms, goethite and magnetite were the major Fe minerals generated (Fig. 1 and 2); formation of these minerals was not observed in Dead or No Cell controls. Additional biological measurements identified an increase in biomass that correlated with an accumulation of total Fe(II), again only in Live treatments. The increase in *mtr* transcripts in FLT treatments supports that the process is biologically mediated (Fig. 3). Secondary Fe minerals, while detectable by flight-ready methodologies such as XRD, are ambiguous as biosignatures since they can also be formed from abiotic processes (55). However, detecting additional biosignatures distinguishable from abiotic backgrounds (68) colocalized with Fe mineralogy would strengthen biogenic claims. Thus, we examined concomitant organic biomarkers associated with *Shewanella* DIR targeting soluble and volatile biosignatures that persisted during the generation of goethite and magnetite.

### Riboflavin

Flavins, including riboflavin, flavin mononucleotide (FMN), and flavin adenine diphosphate (FAD), are electron shuttles ubiquitous across all domains of life (69, 70). During DIR, flavins can be secreted to mediate electron transfer to external terminal acceptors; electrons are shuttled from membrane-bound cytochromes to flavins, which, in turn, reduce the external acceptor (71). Flavins increase the extracellular pool of available electron acceptors, as other modes of electron transfer require direct contact for effective transfer. Many species of Fe(III) reducing bacteria, including *Shewanella* spp., utilize riboflavin as an extracellular electron shuttle (43, 72, 73). Thus, detection of riboflavin in conjunction with reduced Fe mineral deposits is a robust concomitant biosignature for DIR than either alone. In BF02_Schw microcosms, we detected riboflavin via UHPLC-MS at higher abundance in FLT treatments (Fig. 4). In addition, riboflavin appeared to be more abundant in FLT supernatant following 8 weeks of incubation compared to 2 weeks, which could be due to higher production or accumulation within the supernatant over time.

Linking riboflavin to life detection is not unprecedented. However, several caveats should be considered. Case et al. (74) and Wallace et al. (70) proposed riboflavin, FMN, and FAD as useful biosignatures due to their intrinsic pigmentation and autofluorescence. In high Fe environments, FMN and FAD are highly sorbed to Fe (hydr)oxides (75, 76), though they may still be detected via spectral methodologies (e.g., Raman spectroscopy; 77). Riboflavin is highly light sensitive (78) and would likely degrade quickly at the Martian surface. Still, certain environmental conditions increase the stability of riboflavin, such as low temperature, inclusion into a mineral matrix, or shielding from UV, which could include a dust layer, presence at depth, or localization in caves. While structurally different and more stable, niacin, a compound with similar redox function, has been detected in meteorites (79). It is possible that extracellular redox shuttles involved in mineral reduction, such as we observed with riboflavin, can colocalize with transformed mineral products and, under the right environmental conditions, be preserved.

### Porphyrins

Heme cofactors are found throughout all domains of life and are composed of a cyclic tetrapyrrole with an Fe center (80). Heme is essential to the function of bacterial DIR machinery, which utilizes heme cofactors to transfer electrons through the membrane. In addition to their ubiquity, the molecule’s porphyrin core is quite stable and has the potential to remain in the rock record for billions of years as geoporphyrins (81). During diagenesis, side chains and metal complexes become altered while retaining characteristic porphyrin structures (82). Terrestrially, some of the oldest known geoporphyrins, proposed as fossilized remnants of chlorophylls or other Mg-bearing porphyrins, were identified in ~1.1 Ga shale deposits (83). Geoporphyrins uncovered in conjunction with reduced Fe could have originated as heme and therefore serve as biomarkers for ancient DIR or similar metabolisms.

In *Shewanella*, heme is an integral cofactor in four of five proteins in the Mtr complex (84, 85). Transcripts associated with heme biosynthesis were significantly higher in FLT microcosms compared to LT microcosms (Fig. 3), potentially due to greater Fe availability. However, we were unable to detect heme in microcosm supernatant via UHPLC-MS. Heme is associated with membrane-bound proteins, and it is unlikely that heme would be present at detectable levels within the supernatant. Confirmation of heme in BF02_Schw microcosms would likely require extraction before analysis. Detection and characterization of terrestrial geoporphyrins similarly require extensive sample preparation (86), and thus identification of Martian geoporphyrins would most likely require a sample return mission. In addition, challenges may arise in determining the biogenicity of such compounds, as porphyrins have been detected in carbonaceous chondrites (87) and have several plausible mechanisms for abiotic synthesis (as discussed by 88). Regardless, heme, colocalized with reduced Fe-bearing signatures, would strengthen the presumption of microbial DIR.

### Thiosulfate

Sulfates have been discovered in various locales on Mars (e.g., 89) and likely originated during the late Noachian to Hesperian (90). Both thiosulfate oxidation and reduction have been proposed as a viable metabolism for life in late Noachian Mars (91–93). *Shewanella* spp. are known to utilize thiosulfate as both an electron donor (94) and acceptor (53). In addition, Zhu et al. (95) showed that soluble S(-II) from thiosulfate reduction reacts with Fe(II) to form FeS surfaces on both cells and insoluble Fh, which greatly accelerates the rate of electron transfer. The high abundance of sulfates on the surface of Mars may therefore have played a key role in early Martian DIR by enhancing the rate of Fe reduction. In our microcosms, we found that Fh reduction rates increased when incubated with thiosulfate. This process relies on the expression of the terminal reductase-encoding *psr* pathway, which was significantly more transcribed in FLT microcosms (Fig. 3). These data support co-reduction of Fh and thiosulfate and possible generation of FeS minerals, though we were unable to conclusively identify FeS species through our methodologies. Reduction of thiosulfate by *Shewanella* spp. may also lead to production of volatile sulfur compounds (e.g., H_2_S [96]) or sulfur-containing VOCs, which could serve as useful indicators of extant Fh and sulfur metabolism on Mars.

### DMS

The production of differentially abundant VOCs near mineral deposits may function as indicators of extant microbial activity. Microbial VOCs have a high potential for use as biosignatures and can be detected through a variety of methodologies, including local sampling with mass spectrometry or remote sensing via spectral analysis (97). Of VOCs identified in BF02_Schw microcosms, DMS has likely drawn the most astrobiological interest (98, 99), recently gaining media attention for its possible detection on the exoplanet K2-18 b (100, 101). Terrestrially, DMS is largely produced from the breakdown of the metabolite dimethylsulfoniopropionate (DMSP) and is the largest source of biogenic sulfur in the atmosphere (102, 103). While present in relatively high concentrations in marine environments, DMSP production and metabolism are restricted to specific, typically photosynthetic, taxa (104). DMS is also produced microbially through the methylation of methanethiol (105–108). Although this process has long been understood, its potential importance in biogeochemical cycling of DMS may be understated, particularly in soil and sediment environments.

Because it is not linked with abiotic processes on Earth and is potentially detectable via interstellar spectroscopy, multiple studies propose DMS as a possible indicator of an inhabited planet (99, 109). DMS is readily degraded in Earth’s atmosphere and thus does not reach concentrations detectable via interstellar means (99, 110). However, on planets without O_2_-rich atmospheres and lower levels of UV radiation, DMS may accumulate to a greater abundance, and therefore be within spectroscopically detectable ranges. On smaller scales, DMS can be detected in trace amounts using mass spectrometry, such as with the proton-transfer-reaction mass spectrometry (PTR-MS) used in this study. Historically, sea-to-air transfer of DMS has been considered a relatively small proportion of oceanic production of DMS and may represent “leakage” from a biological system (111–113). Thus, even if atmospheric DMS concentrations are not sufficiently high to be detected on planetary bodies, “hot spots” with higher DMS concentrations associated with aquatic features may be detectable by surface missions.

On Mars, organosulfur compounds were discovered in Gale Crater mudstone via pyrolysis-GC-MS and evolved gas analysis using the SAM instrument (114) with DMS or its isomeric equivalent ethanethiol detectable in the pM range, though laboratory experiments suggested that ethanethiol was the primary isomer (115). In BF02_Schw microcosms, DMS was detected in greater abundance in FLT microcosms following an 8-week incubation (Fig. 5). While not detected at the 2-week time point, increased transcript abundance for *dms* gene clusters in FLT microcosms suggests that DMS was produced in 2-week incubations, though not at detectable levels (Fig. 3). The *dms* pathway encodes terminal reductases that are utilized by *Shewanella* spp. during respiration of DMSO, leading to the production of DMS (54); *dms* genes in different bacteria have been implicated in other reactions, such as reduction of methionine sulfoxide (116) and nicotinamide-, pyrimidine-, and adenine-N-oxide (117). While these data indicate that DMS production by BF02_Schw under our FLT conditions occurs, it remains unclear what the specific mechanisms are and if both abiotic and biotic processes are involved. DMS may also form by yet-unknown abiotic processes; recent evidence for DMS was identified on comet 67P/Churyumov-Gerasimenko (118). So, while detection of DMS would not provide unequivocal evidence for life, its detection in association with other mineral or chemical biosignatures on Mars may guide more targeted sampling for life detection missions and sample return.

### Halogenated compounds

On Earth, chloromethane and dichloromethane are produced as a result of both biotic and abiotic processes, such as the nonspecific degradation of organic matter, often by haloperoxidases (119, 120). Halogenated compounds, including chlorate and perchlorate, may be reduced by microorganisms, including *Shewanella* spp., leading to the generation of O_2_ and Cl^−^ (121, 122). Halogens can also be incorporated into microbial natural products, such as antibiotics (119), which, due to their complexity, are strong indicators of biological synthesis. During preliminary PTR-MS experiments performed in complex media, we detected the production of chloromethane by BF02_Schw when grown under both aerobic and anaerobic conditions, as well as bromomethane during aerobic incubation (see supplemental material; Fig. S15). While the *Viking* GC-MS identified chloro- and dichloromethane, experiments performed using terrestrial analog soils suggest that these compounds likely formed from reactions of organic matter with soil perchlorates, which are abundant on Mars (123). Thus, volatile organohalides alone would be challenging to interpret in a Martian setting.

Interestingly, we were also able to detect transcription of a putative tryptophan halogenase (*prnA*) in minimal media treatments. Flavin-dependent halogenases have been shown to add halogen atoms to a variety of substrates (119). Of transcripts annotated as flavin-dependent halogenases, only *prnA* contained conserved binding sites for tryptophan and FAD (as reviewed in reference 118); Fig. S16) and had high structural homology to characterized tryptophan halogenases (Fig. S17; Table S6). While *prnA* is likely not associated with the observed chloro- or bromomethane volatiles, halogenation of organic compounds by BF02_Schw appears to occur under all examined conditions. Considering the hypothesized global distribution of halogenated compounds across the surface of Mars (124), these results suggest that further investigation targeting the production of halogenated compounds in Martian contexts is certainly warranted.

### Implications for future *in situ* planetary investigations

This project employed types of analyses that have been or may be deployed on Mars, including MS, XRD, VNIR, FTIR, Raman, and Mössbauer spectroscopies (Table 2). Both orbital and *in situ* missions to Mars have long supported the presence of water on early Mars (1–3, 125), and given predicted conditions, DIR is a reasonable metabolic strategy for any early Martian life (46). The results of this study have the potential to be directly applied to interpreting data from past, current, and future missions to Mars. For example, a “*Viking 2.0*” mission for the detection of DIR on Mars utilizing a specific suite of instruments and sample incubation capabilities could provide strong lines of evidence beyond the abilities of current missions. Instrument selection for a reimagined life detection mission would likely differ from combinations employed previously. For example, despite the utility of XRD in the identification of Fe^2+^ species in Gale Crater (4, 61), we were unable to identify mineralogical changes via XRD (Fig. 1C; Fig. S4). Similarly, while Raman spectroscopy has the potential to detect stronger organic biosignatures such as porphyrins, no identifiable changes were observed between our biotic and abiotic treatments (Fig. S8). Mass spectrometry approaches have been utilized several times on Mars and have detected possible organic biosignatures *in situ* (e.g., 38); future microcosm experiments should explore the detection of organics via similar GC-MS instrumentation. Several successful methods utilized in this study, such as TEM and LC, require significant development to be viable in remote settings. Currently, these will require either a sample return or a crewed mission to perform nuanced incubation-based studies to increase the chance of detection. It must also be noted that while the individual biosignatures detected in this study would not constitute a confirmation of life, the discovery of co-localized reduced Fe, molecules utilized in electron shuttling, and volatile byproducts of Fe-S metabolism increases the strength of evidence and would certainly warrant a more detailed study.

**TABLE 2:** Summary of detected biosignatures

Instrument/method	Detected biosignatures	Relative strength of biosignature for DIR detection	Examples of Mars-relevant instrumentation	Other proposed instrumentation
XRD	None—bulk Fe reduction not detected	NA^*b*^	MSL ChemMin (34); Mars 2020 PIXL XRF (35)	–^*a*^
VNIR	Reduced Fe (magnetite)	Lower (abiotic origin?)	MERs: Pancam (26); MRO: CRISM (28); *Mars Express*: OMEGA (29) Mars 2020: SuperCam (27)	–
FTIR	Reduced Fe (magnetite, geothite)	Lower (abiotic origin?)	–	Broadly comparable to other IR methods
Mössbauer	Reduced Fe (geothite, magnetite)	Lower (abiotic origin?)	MERs: MIMOS II (33)	–
Raman	None—overwhelmed by Fe fluorescence	If detection of porphyrins, higher	Mars 2020: SHERLOC (36), SuperCam (27)	–
TEM + SAEM	Reduced Fe (magnetite, geothite)	Lower (abiotic origin?)	Likely utilized upon sample return	–
UHPLC-MS	Riboflavin	Higher—likely degrades quickly at surface	No LC; *Viking*: GC-MS (21); MSL: SAM (37)	Detectable via fluorometry (70, 74)
PTR-MS	DMS	Higher—supports combined Fe-S metabolism	*Viking*: GC-MS (21); MSL: SAM (37)	Detectable via spectroscopy (99, 109)
	Halogenated compounds	Intriguing as biosignature, links to DIR uncharacterized	*Viking*: GC-MS (21); MSL: SAM (37)	–

^*a*^ –, methods have not been deployed to Mars or other astrobiologically relevant instrumentation has not been proposed.

^*b*^ NA, not applicable.

### Conclusion

Microorganisms isolated from extreme environments can be utilized to validate signals from astrobiologically relevant instrumentation, test how biosignatures vary under a range of environmental conditions, and potentially discover novel biosignatures. Life on Mars may have occurred early in its history under warmer, wetter conditions. In these early ecosystems, Fe reduction may have been a dominant primitive metabolism, facilitated in part by the Martian sulfur cycle, leaving biologically altered Fe minerals in the rock record. When grown in anaerobic laboratory microcosms, BF02_Schw mediated the reduction of Fh to goethite and magnetite, production of VOCs including DMS and chloro- and bromomethane, and synthesis of the electron shuttle riboflavin. Simultaneous detection of multiple biosignatures associated with mineral transformations on another planetary body would provide far more compelling evidence of life. However, detection of biosignatures such as volatiles or flavins with flight-ready instrumentation likely would require sample incubation for long-term headspace monitoring if utilizing methods such as PTR-MS. While not within the capabilities of current surface missions, future missions may endeavor to develop analyses that target biosignatures that utilize incubation or extraction methodologies in high-priority sampling locations or instrumentation to detect ambient volatiles to target sampling efforts for *in situ* experiments or even sample return. These methodologies are reminiscent of the experiments on the *Viking 1* and *2* landers and highlight the promise of deploying similar payloads, albeit with a modern approach.

## MATERIALS AND METHODS

### Culture conditions

*Shewanella* sp. strain BF02_Schw was isolated from Blood Falls, Antarctica, in 2002 (described in reference 47). For all experiments, strain BF02_Schw inocula were grown in Difco Marine Broth 2216 (BD) at 15°C to the stationary phase before transfer to a marine minimal medium. All experimental incubations were performed at 4°C. Marine minimal medium broth contained the following chemicals per 1 L of Milli-Q water: sodium chloride (333.0 mM), magnesium chloride (92.43 mM), sodium sulfate (22.81 mM), calcium chloride (16.22 mM), potassium chloride (7.377 mM), sodium bicarbonate (1.905 mM), potassium bromide (672.3 µM), strontium chloride (127.5 µM), boric acid (355.8 µM), sodium metasilicate (14.1 µM), sodium fluoride (57.2 µM), and ammonium chloride (20.0 µM). The medium was supplemented with 1 mL L^−1^ of MD-TMS Trace Minerals Supplement (ATCC) and 2 g L^−1^ of SC Amino Acids Mixture (MP Biomedicals) and dispensed into serum vials. Vials were thoroughly flushed with nitrogen gas (Ultra High Purity Grade; Airgas), capped with butyl stoppers, and crimp-sealed before autoclaving at 121°C for 20 minutes. Following autoclaving, each container was supplemented with a filter-sterilized disodium phosphate solution (29.8 µM). Sterile solutions of sodium lactate (5 M) and sodium thiosulfate (2.5 M) were prepared using Milli-Q water and autoclaved for 20 min at 121°C and added to final concentrations of 30 mM and 10 mM, respectively, in relevant media (see Table 1). Fh (1M) was prepared following reference 126 and sterilized by heating to 90°C for 20 minutes and cooling to 4°C twice. Fh sterility was confirmed via plating on Difco Marine Agar 2216 (BD), and added via syringe to media at a final concentration of 100 mM in Fh-containing media (Table 1). BF02_Schw inocula were pelleted and washed three times with the basal salt solution (i.e., no Fh, thiosulfate, lactate, or amino acids) before inoculation in serum vials at a 1:50 dilution. “Dead” inocula were prepared by autoclaving an aliquot of washed cells for 20 minutes at 121°C. All vials were wrapped in aluminum foil to prevent photodegradation of Fh. Analyses were performed following either 2 or 8 weeks of incubation; due to logistical constraints around instrumentation, exact sampling times varied slightly between experiments (13–14 days for the 2-week incubations, 56–60 days for the 8-week incubations). For each time point, cultures were thoroughly mixed before 2 mL aliquots were drawn via syringe, with 1 mL aliquoted for plate counts and insoluble Fe concentration, and 1 mL passed through a 0.2 µm syringe filter for soluble Fe concentration, as described below. Unfiltered aliquots were diluted using marine minimal medium basal salts, plated on Difco Marine Agar 2216 (BD), and incubated at 15°C for 1 week for CFU counts. Both filtered and unfiltered aliquots designated for Fe were diluted 10-fold in 0.5 N HCl in acid-washed glass vials and stored at 4°C until analysis.

### Colorimetric iron concentration

Fe concentration was determined via colorimetric assay using the FerroZine Iron Reagent (Thermo Scientific). Briefly, a 0.02% FerroZine solution was prepared in 1.2% HEPES buffer. A reducing buffer was prepared by adding hydroxylamine to the FerroZine solution (final concentration 1%). Unfiltered samples were diluted 1:10 and aliquoted into two 96-well plates in triplicate, followed by the addition of either FerroZine solution or the reducing buffer, and incubated for 15 minutes, then the absorbance was measured at 562 nm using a Synergy HT plate reader (BioTek) and compared to a standard curve of ferrous ammonium sulfate and statistically compared in R v4.3.2 (127) using linear mixed-effect models to account for biological and technical replicates through the lmerTest package v3.1-3 (128). Models were assessed via ANOVA and Tukey’s all-pair comparison using the car v3.1-2 (129) and multcomp v1.4-25 (130) packages, respectively. Features were considered statistically significant if *P* ≤ 0.05.

### Mineralogy

Incubations designated for mineral analyses were performed in 50 mL volumes as described above for Fh containing treatments (i.e., FLT, FL; Table 1). At each time point, cultures were heat-killed at 40°C for 3 hours to avoid abiotic transformation of Fe species and shipped at 4°C for analysis.

#### X-ray diffraction

XRD analyses used a Rigaku Smartlab II SE XRD with Cu Kα radiation in Bragg-Brentano geometry. Scans were performed as 5°–80° (0.01° step size) or 2°–80° (0.02° step size) 2θ scans at a rate of 2° min^−1^. Both baseline correction and mineral identification were performed using Smartlab Studio II software (Rigaku) with a licensed International Centre for Diffraction Data PDF 2 database (2019 version [131]). Both profile and peak data were used to identify and quantify mineral phases via the Smartlab Studio II XRD plugin. Each pattern was adjusted to a tight B-spline baseline as well as an adjusted baseline to account for amorphous baseline swell.

#### FTIR spectroscopy

FTIR spectra were collected using a Bruker ALPHA FTIR spectrometer with a platinum diamond ATR attachment. Spectra were collected at a 4 or 8 cm^−1^ resolution from 360 to 400 cm^−1^, and final spectra were the result of averaging 128 scans per sample. Bruker’s OPUS software 7.3 was utilized for continuum removal via concave rubber band correction. Reference spectra include previously collected spectra (132) and spectra from the USGS Spectral Library (133).

#### VNIR spectroscopy

VNIR spectra were obtained on an ASD Fieldspec 4 Max spectrometer in bidirectional geometry. The spectrometer was equipped with an Oceans Optics HL-2000 light source directed down a 1,000 µm Si optical fiber at 30° and an 8° foreoptic for perpendicular collection. Prior to measurement, samples were leveled without packing in matte-black painted sample cups, and spectra were collected without ambient illumination. Spectra were obtained as 3 × 240 136 ms integrations and referred against Spectralon standards, USGS references (133), and previously collected spectra (132).

#### Raman spectroscopy

Raman spectra were obtained using a Bruker BRAVO spectrometer with excitation lasers at 758 and 852 nm and a 300–3,200 cm^−1^ range. Samples were analyzed through the bottom of one dram glass vials, and resulting spectra were averaged over 100–1,000 ms integrations. Baseline correction was performed using the rubber band algorithm as implemented at http://nemo.mtholyoke.edu. Spectra were compared to the RRUFF database (134) and previously collected spectra (132).

#### Mössbauer spectroscopy

Mössbauer spectroscopy was performed using a Web Research (now See-Co) W302 Mössbauer spectrometer at 295, 220, 150, 80, and 4K employing a Janus closed-cycle He compressor at <295 K. Samples were dried and ground lightly with sugar in secured plastic washers backed with Kapton tape. Each 1,024-channel spectrum was folded about the midpoint, and an α-Fe foil was used for calibration via the WMOSS4 program (135). Spectra fitting was performed using the Mexfield program (University of Ghent).

#### Transmission electron microscopy

TEM images with SAED data were collected on the Johns Hopkins University Materials Characterization and Processing Center’s Thermo Fisher TF30 instrument operating at 300 kV with an EDAX windowless silicon drift energy dispersive X-ray (EDX) detector. Conventional imaging, high-resolution lattice imaging (HRTEM), SAED, scanning transmission electron microscopy (STEM), bright-field (BF), high-angle annular dark-field (HAADF) imaging, and STEM EDX maps were collected for selected samples. Images of the *Shewanella* cells were acquired at Mount Holyoke College using a Phillips CM 100 TEM equipped with a 40–100 KV tungsten filament, single-tilt goniometer stage, and an AMT digital camera.

### RNA extraction and sequencing

Cultures for RNA sequencing were incubated in 50 mL volumes as described for the mineralogical analysis, with LT and FLT treatments. At each time point, cultures were pelleted (2,500 × *g* for 30 min), immediately frozen on dry ice, and stored at −80°C until extraction. RNA extractions were performed using the Invitrogen TRIzol reagent following the manufacturer’s protocol with the following amendments: pellets from FLT microcosms were thawed in 3.75 mL PB buffer (112.87 mM Na_2_HPO_4_ + 712 mM NaH_2_PO_4_) and 1.25 mL TNS buffer (500 mM Tris-HCl +100 mM NaCl +10% wt/vol SDS) before centrifuging at 2,500 × *g* (1 min) to pellet insoluble Fe, as described by 136. This step was repeated twice more, until no visible Fe pellet remained, followed by the addition of TRIzol reagent. Pellets without Fh were thawed directly in TRIzol. Following extraction, the remaining genomic DNA was removed via DNase I treatment (Invitrogen), and samples were concentrated via ethanol precipitation. Removal of DNA was confirmed via PCR, and RNA was quantified via both fluorometry and automated electrophoresis before shipment to the University of Maryland Genomics Core for cDNA library preparation and sequencing via Illumina NovaSeq 6000 (2 × 100 bp).

### DNA and RNA sequence analysis

Returned RNA sequences were aligned to the published strain BF02_Schw genome (48) using bowtie2 v2.2.5 (137) using default parameters. Count files were generated using samtools v1.9 (138) and aniv’o v5.5 (139). Reads were assembled into transcripts using StringTie v2.1.7 (140), and transcripts were quantified using Salmon v1.10.1 (141). Differential expression was analyzed using DESeq2 v1.34.1 (142) in R v4.3.2 (127). Transcripts were considered significantly more or less abundant if log_2_ fold change (LFC) was ≥1 or ≤ −1 , and the adjusted *P* value (Benjamini-Hochberg adjusted Wald test; *P*_adj_) was ≤0.05.

Both genes and transcripts were annotated using three methods. Initial open reading frame identification and sequence annotation were performed using Prokka v1.14.5 (51). Open reading frames were placed within pathways using the KEGG database (143) through GhostKOALA v2.0 (144). Specific gene targets were identified using BLAST v2.15.0 (145) against both in-house curated databases and the UniProt database (146). Only sequences with evidence at the protein level were considered for database inclusion. Further investigation of putative function for halogenase genes involved alignment of BF02_Schw sequences to characterized flavin-dependent tryptophan halogenase sequences to compare known conserved binding domains using Geneious v6.1.8 (147). Proteins with hypothetical binding domains were computationally folded using ColabFold v1.5.5 (148) and compared to databases of characterized protein structures using the Dali web server (149). The alignment figure was generated with ESPrint v3.0 (150).

### Soluble metabolites

Soluble metabolites were identified in pooled supernatant via UHPLC-MS. Supernatant was sourced from the transcriptomics experiment and included FLT and LT treatments; supernatant from the 8-week LT microcosms was not collected. Supernatant was extracted in an equal volume of methanol (LC-MS grade) via sonicating in a water bath (10 min) and centrifugation. Extracts were analyzed on a Vanquish Horizon UHPLC system and Q-Exactive HF-X Hybrid Quadrupole-Orbitrap MS (Thermo Scientific). The LC instrument was configured with an HSS T3 C18 column (Waters, 1.8 µm, 2.1 × 100 mm) using a VanGuard FIT precolumn and a VH-D10-A UV detector. Samples (2 µL) were injected using a flow rate of 0.5 mL min^−1^ into a prewarmed column (40°C). UHPLC separation was performed using the following parameters: 2% acetonitrile: 98% water with 0.1% formic acid for 1 min, 2%–40% over 4 min, 40%–98% over 3 min, 98%–2% over 0.2 min, and 2% for 2 min. UV–Vis data were collected at 534 nm. Electrospray ionization occurred in probe position D using the following conditions: 40 sheath gas flow, 8 auxiliary gas flow, 1 sweep gas flow, 3.5 kV spray voltage, 380°C capillary temperature, 50 radiofrequency funnel level, and 350°C auxiliary gas temperature. Resulting spectra were aligned using MS-DIAL v5.2.240218.2 (151), and peak filtering and analysis were performed using MPACT v23.05.15 (152). Solvent blank filtering was applied using a group parsing threshold of 0.01, and a minimum reproducibility threshold of 0.5 median coefficient of variation among technical replicates was utilized. Presence or absence of features was determined using a 0.05 relative abundance threshold. Putative riboflavin peaks were compared to a known riboflavin standard (Sigma-Aldrich).

### Volatile organic compounds

Two different techniques were used to assess the VOCs produced by BF02_Schw: an Ionicon PTR-TOF-1000 Mass Spectrometer (PTR-MS) operating in the hydronium ion mode to survey the suite of VOCs present with *m/z* ratios ranging from 20 to 250, and a Bay Instrument Membrane Inlet Mass Spectrometry (MIMS) system to examine the production of specific compounds from precursors of known masses.

Incubations designated for VOC surveys were performed on 100 mL aliquots of experimental treatments in acid-cleaned 1L Schott media bottles and sealed with a GL45 screw cap incorporating a butyl rubber septum. This ratio of sample-to-container size was chosen to maximize headspace volume for sampling. Sterile bottles thoroughly flushed with Ultra High Purity nitrogen were utilized as additional headspace controls.

Upon instrumental analysis using the PTR-MS, samples were connected to the mass spectrometer via a sampling manifold with two gas lines. Each gas line was fitted with syringe needles that were inserted through the septum for sampling. The needle from the inflow line was placed close to the surface of the sample, and the outflow needle was positioned near the top of the bottle, close to the septum. Sample headspace was then flushed past the mass spectrometer’s inlet with zero-grade nitrogen gas at a rate of 80 mL min^−1^, with the mass spectrometer subsampling the gas stream at a rate of 50 mL min^−1^. Mass spectra were acquired every second for 10 minutes and averaged to provide a single mass spectrum for each sample. Subsequent baseline and peak detection was carried out using Progenesis MALDI (Nonlinear Dynamics, Newcastle upon Tyne, United Kingdom). Differential abundance was determined using ANOVA in Progenesis MALDI, and identified features were statistically compared in R via ANOVA (car v3.1-2 [124]) and Tukey’s all-pair comparisons (multcomp v1.4-25 [130]) using a 0.05 cut-off for significance.

Production of volatile sulfur compounds by BF02_Schw from specific precursors was investigated using MIMS as per Schanke et al. (153). BF02_Schw cultures were grown in minimal media with the addition of Fh + lactate + thiosulfate. Labeled dimethyl sulfoxide (D_6_-DMSO, 118.6 µM) was added to the medium, and the production of both isotopically and non-isotopically labeled DMS was assessed over 2 weeks.

## Data Availability

The genomic sequence of BF02_Schw was published previously in Boles et al. (48) under NCBI accession number JABKAW000000000, and assembled transcripts from LT and FLT treatments are available under NCBI BioProject PRJNA1261965. Raw LC-MS spectra have been uploaded to Zenodo (https://doi.org/10.5281/zenodo.15389915). Analysis scripts and raw data files are available upon request.

## SUPPLEMENTAL MATERIAL

Supplemental material aem.02528-24-s0001.docx Methods for preliminary VOC experiments, Fig. S1 to S17, and Tables S1, S2, S4, S5, and S6.

Table S3 aem.02528-24-s0002.xlsx Complete transcript table for *Shewanella* sp. strain BF02_Schw grown with and without 100 mM ferrihydrite.

ASM does not own the copyrights to Supplemental Material that may be linked to, or accessed through, an article. The authors have granted ASM a non-exclusive, world-wide license to publish the Supplemental Material files. Please contact the corresponding author directly for reuse.
