# Supplementary material for: Multi-technique characterization of iron reduction by an Antarctic Shewanella: an analog system for putative Martian biosignature identification
Source: Appl Environ Microbiol. 2025 Jul 10;91(8):e02528-24. doi: 10.1128/aem.02528-24 (PMC12366341; doi:10.1128/aem.02528-24)
Supplement: Supplemental material — Methods for preliminary VOC experiments, Fig. S1 to S17, and Tables S1, S2, S4, S5, and S6. [file aem.02528-24-s0001.docx]

**Supplementary Materials for “Multi-technique characterization of iron reduction by an Antarctic *Shewanella*: an analog system for putative Martian biosignature identification”**

**PRELIMINARY VOC EXPERIMENTS**

**Methods**

Preliminary headspace analyses were performed via PTR-MS as described in the main text. Two sets of incubations were analyzed: the first included Live, Dead, or No Cell treatments inoculated in Difco Marine Broth 2216 (BD) under an aerobic headspace flushed with ultra-pure air. The second utilized Live, Dead, and No Cell treatments and were performed in Difco Marine Broth 2216 (BD) supplemented with 100 mM Fh and incubated with an ultra-pure N_2_ headspace.

**Results**

In preliminary Marine Broth incubations, 14 features were identified as significantly higher in aerobic Live treatments, and 5 features were significantly higher in anaerobic Live treatments. Anaerobic features included *m/z* 44.3, 46.25, 48.23, 49.21, 51.17, 62.95, 78.54, 80.5, 92.21, 94.18, 96.13, 109.72, 125.33, and 171.93. The feature with *m/z* 96.13 was putatively identified as bromomethane, 62.95 as DMS, 51.17 as chloromethane, and 49.21 as methanethiol (Fig. S15). Features significantly higher in anaerobic incubations include *m/z* 48.97, 50.97, 62.99, 78.93, and 94.95. Putative identities include DMS (*m/z* 62.99), chloromethane (*m/z* 50.97), and methanethiol (*m/z* 48.97).

**References**

1. Sklute EC, Kashyap S, Dyar MD, Holden JF, Tague T, Wang P, Jaret SJ. 2018. Spectral and morphological characteristics of synthetic nanophase iron (oxyhydr)oxides. Phys Chem Minerals 45:1–26.

2. Kokaly RF, Clark RN, Swayze GA, Livo KE, Hoefen TM, Pearson NC, Wise RA, Benzel W, Lowers HA, Driscoll RL, Klein AJ. 2017. USGS Spectral Library Version 7. 1035Data Series. U.S. Geological Survey.

3. Lafuente B, Downs RT, Yang H, Stone N. 2015. The power of databases: the RRUFF project., p. 1–30. *In* Highlights in Mineralogical Crystallography. De Gruyter, Berlin, Germany.


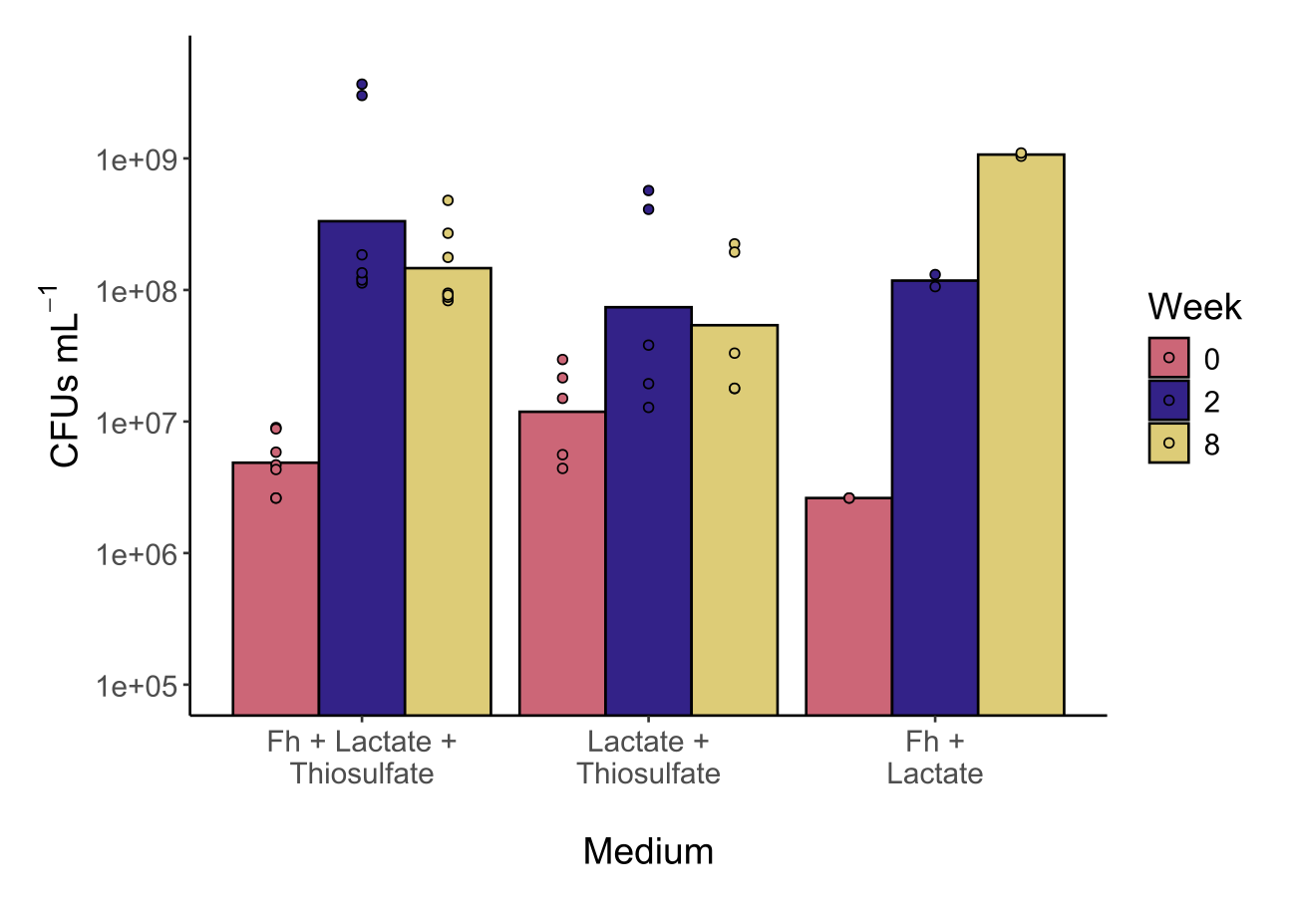


**Fig S1.** Increase in CFUs following two and eight weeks of incubation for all experiments.


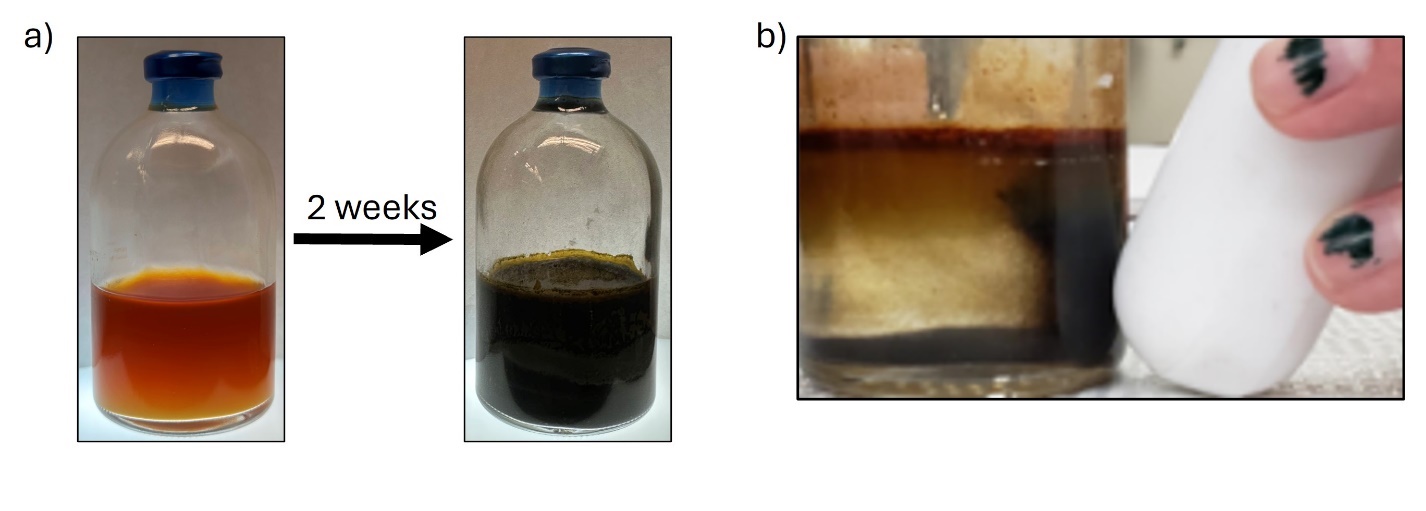


**Fig S2.** Visual transformation of insoluble in Fe following 2 weeks of incubation in Live FLT incubations.


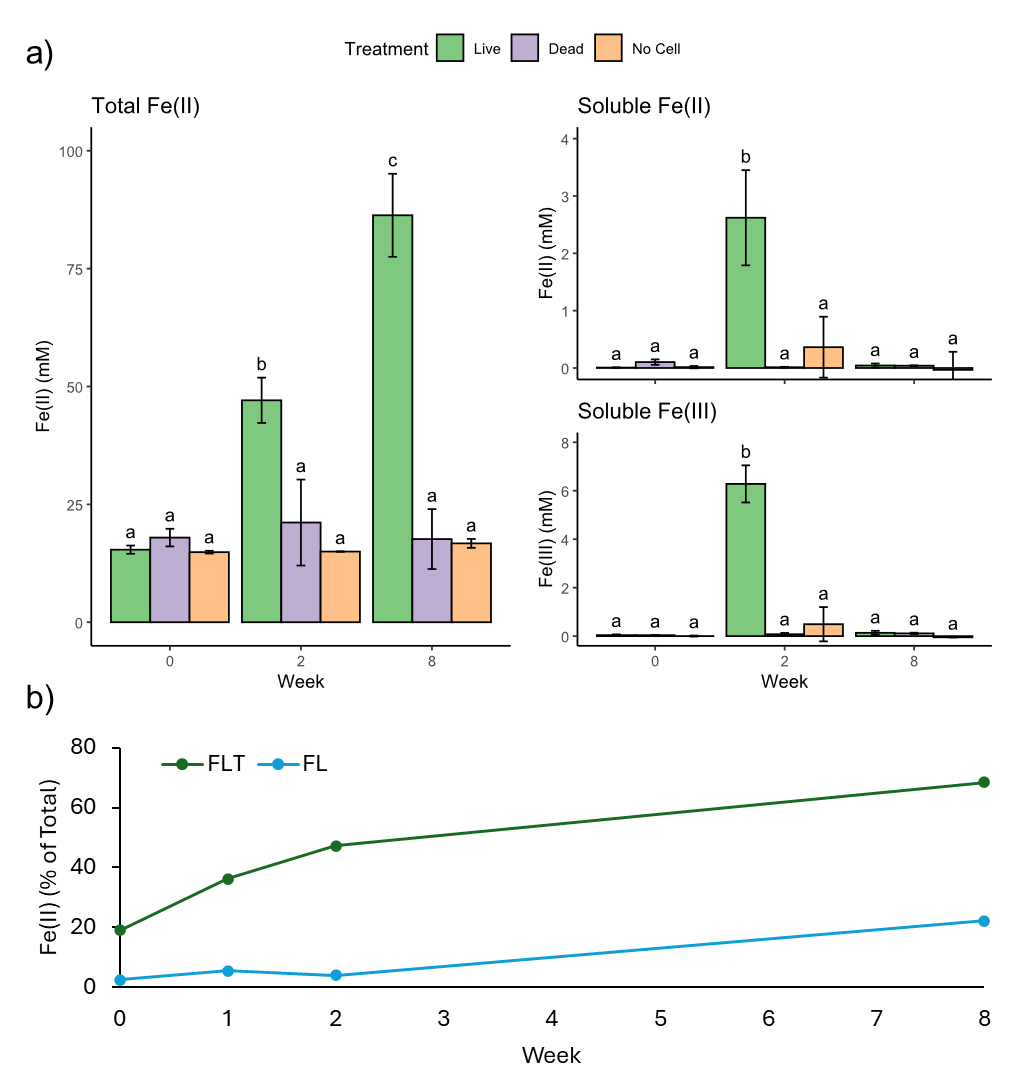


**Fig S3.** Reduction of Fe(III) to Fe(II) as evidenced by FerroZine^TM^ colorimetric assay. All values are normalized to total Fe. (a) Fe(III) reduction in FLT microcosms. Total Fe(II) increased in Live microcosms over the course of incubation (t-test, *** = p < 0.001, ns = not significant). Soluble Fe(II) and Fe(III) increased only on Day 13. (b) Fe(III) reduction in Live mineral microcosms. Treatments containing Fh + lactate + thiosulfate had greater Fe(III) reduction than those without thiosulfate. *ns – not significant*


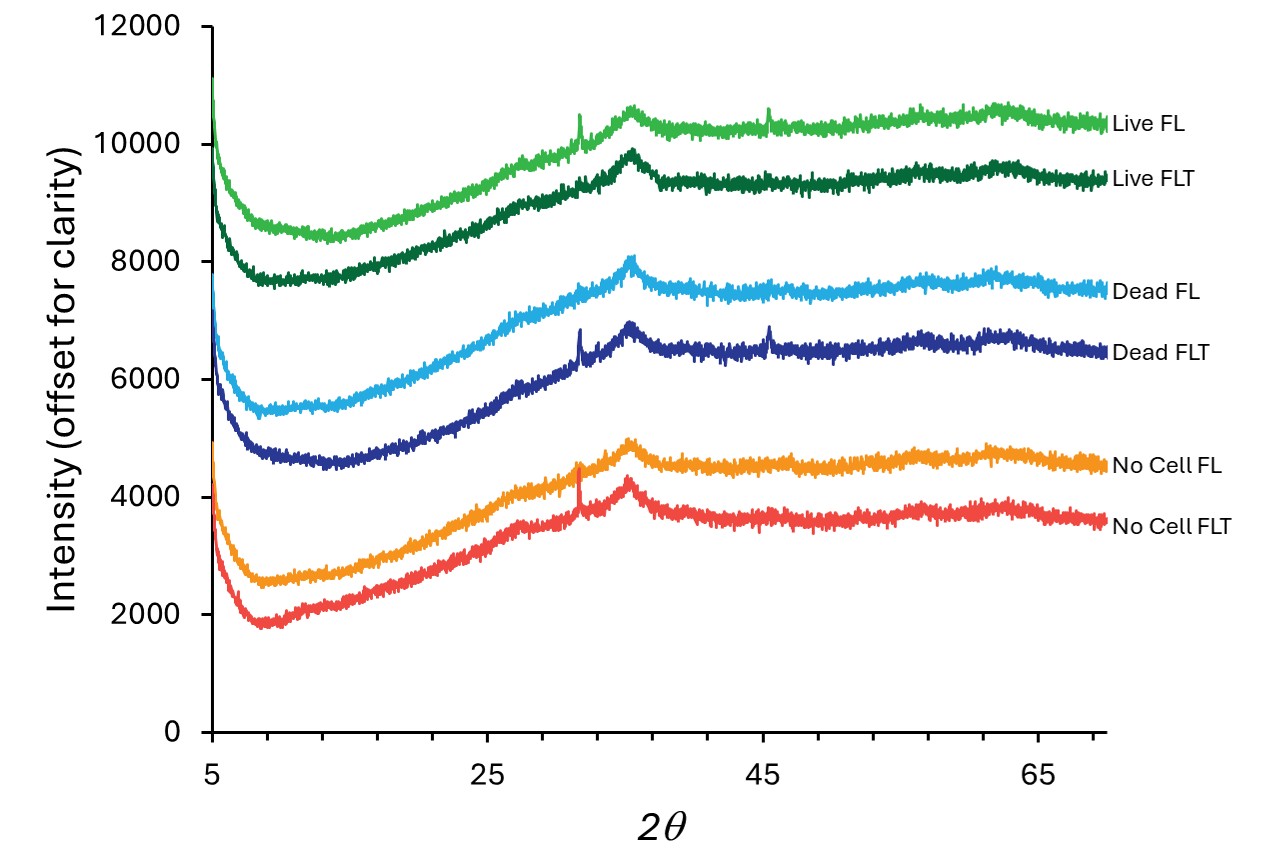


**Fig S4.** XRD spectra collected for all treatments following two weeks of incubation.


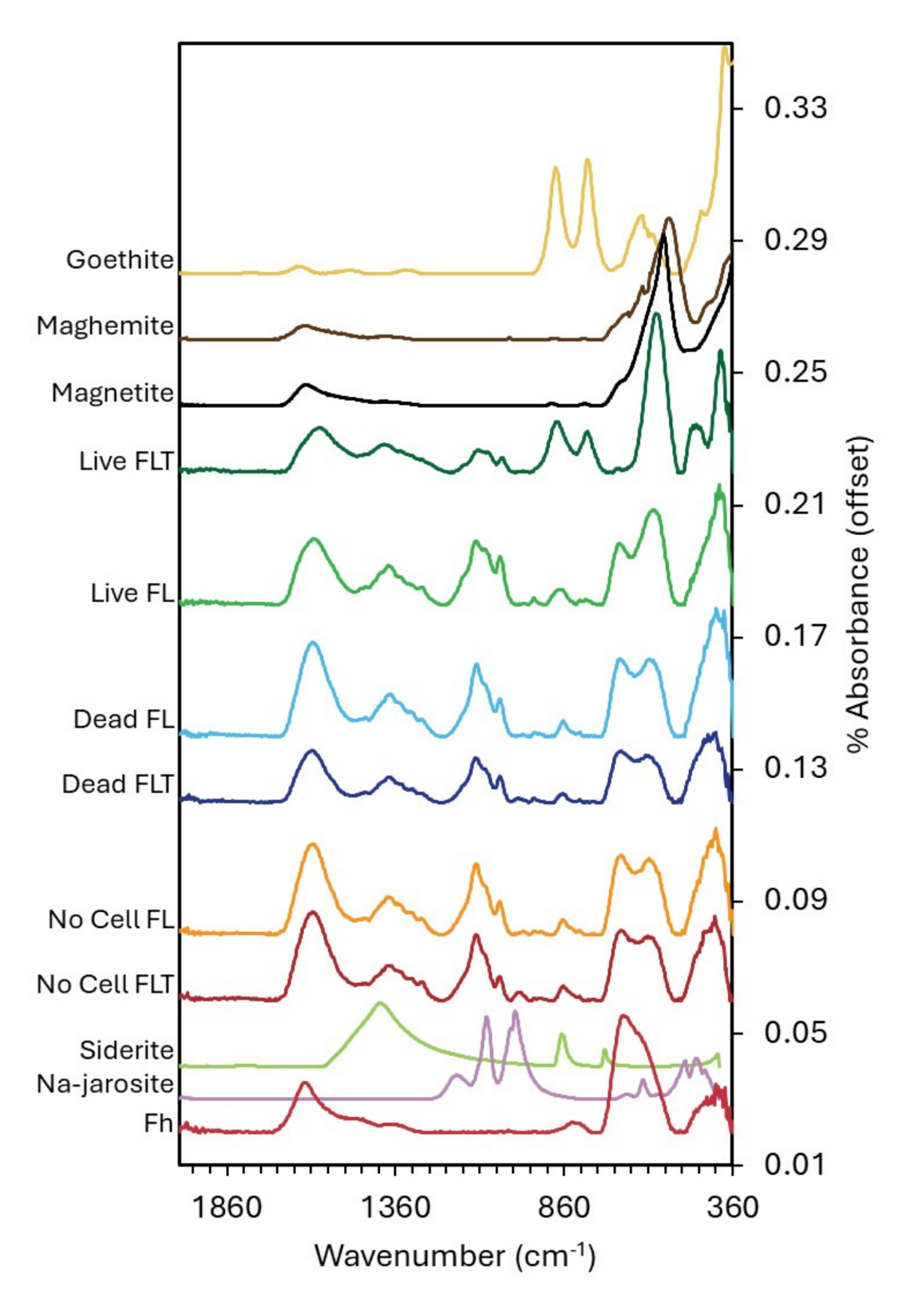


**Fig S5.** FTIR spectra of reaction products and controls for eight-week FLT and FL incubations. Spectra of siderite from the USGS specli 7 (131) and for several nanophase Fe oxides spectra from Sklute et al. (1) are shown for reference.


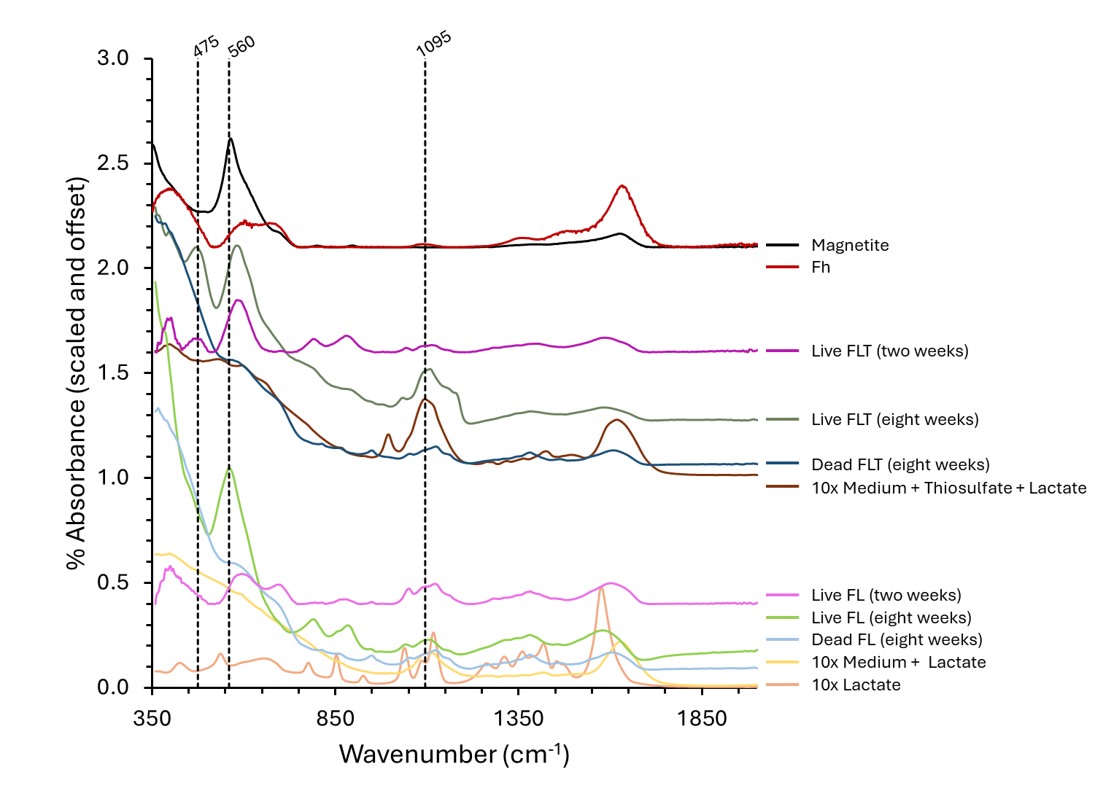


**Fig S6.** FTIR spectra of two- and eight-week Live treatments, eight-week Dead treatments, and unincubated (fresh) 10x concentrated marine minimal medium with lactate, or lactate + thiosulfate (no Fh), in addition to 10x concentrated lactate or thiosulfate. Plot is grouped by experimental condition and scaled and offset for clarity. Magnetite and Fh are shown for reference. Note only reference oxides and two-week incubations have been baseline removed.


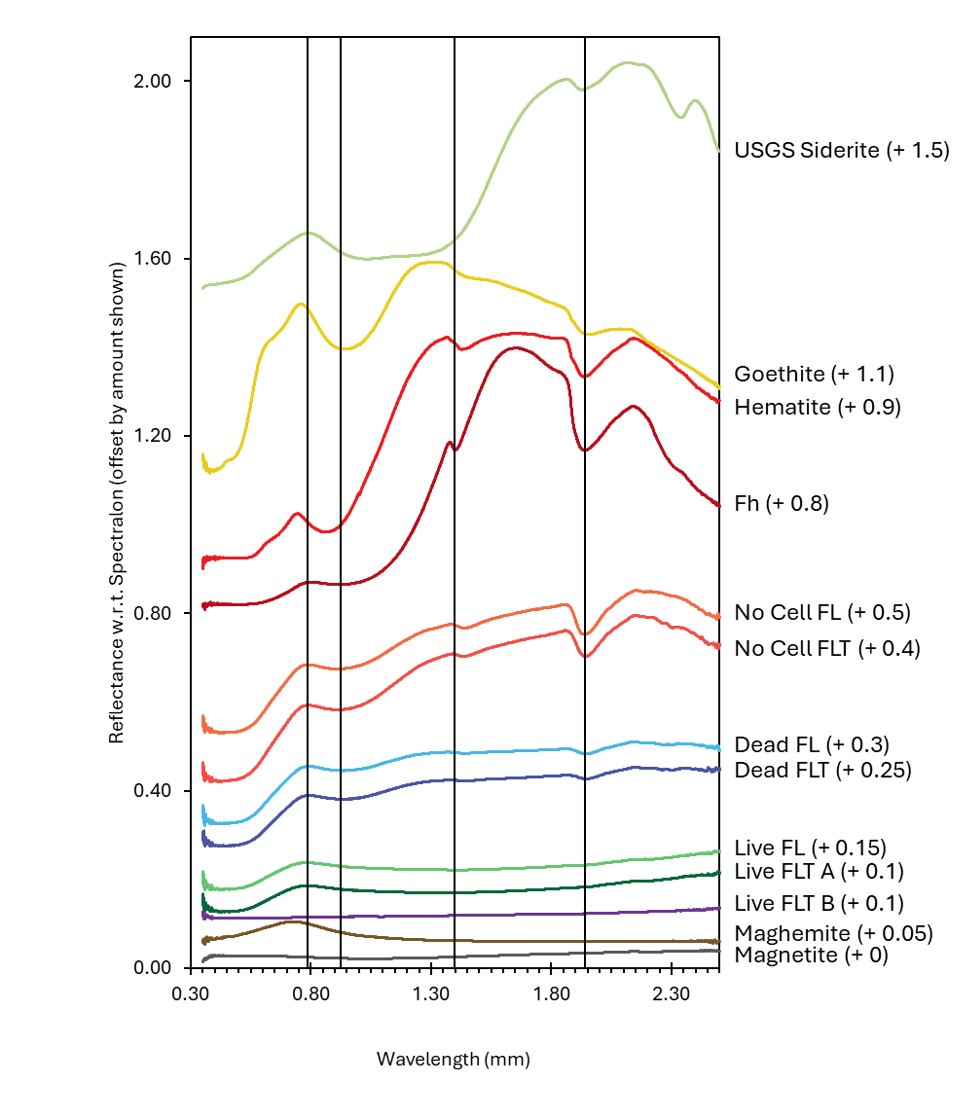


**Fig S7.** VNIR spectra of two-week incubations and controls for experiments with FLT and FL treatments. One replicate of each condition is shown except for the Live FLT treatment, where the products differed spectrally. Spectra of siderite from the USGS specli 7 (2) and for several nanophase Fe oxides spectra from Sklute et al. (1) are shown for reference.

**
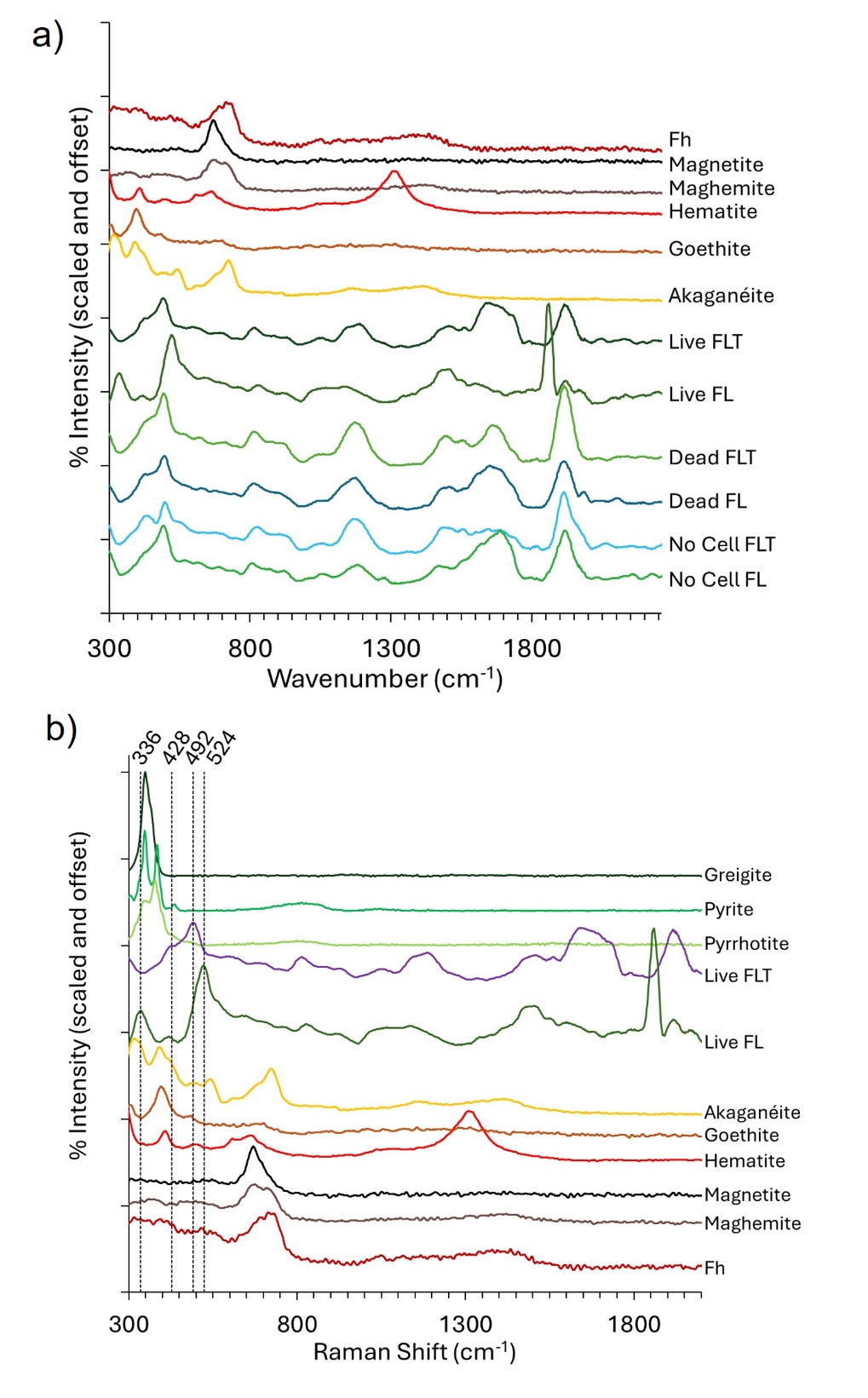
**

**Fig S8.** (a) Raman spectra acquired from two-week mineral treatments compared to oxides from Sklute et al. (1). (b) The Live FL treatment showed some spectral changes on one analysis. That spectrum, along with Live FLT are shown compared to (hydr)oxides from Sklute et al. (1) and RRUFF sulfide samples pyrite R050190, pyrrhotite R606440, and greigite R120103 from the RRUFF database (3).

**
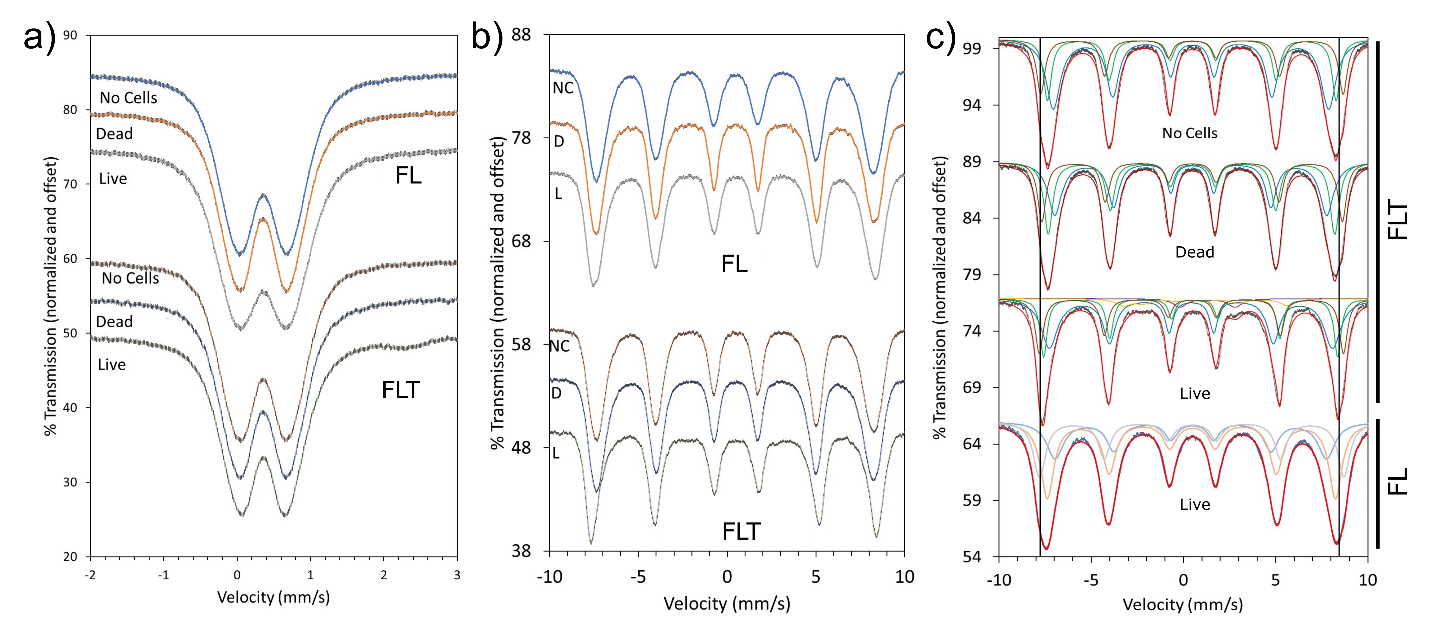
**

**Fig S9.** Mössbauer spectra of two-week treatments acquired at (a) 295K and (b) 4K. (c) Fits of Mössbauer spectra at 4K. All treatments contain Fh.


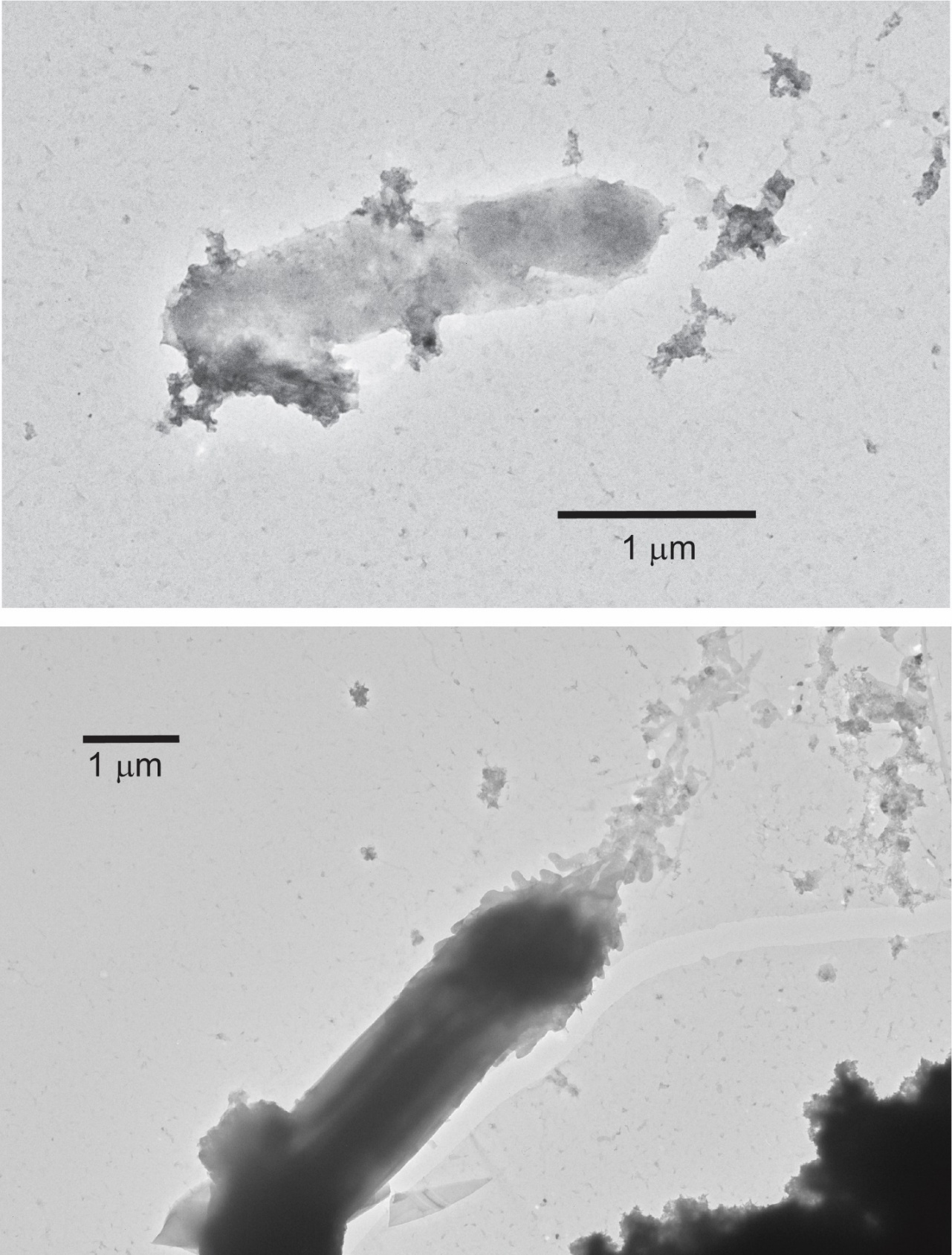


**Fig S10**. TEM images of a BF02_Schw cell associated with Fe oxides in two-week FLT treatments.


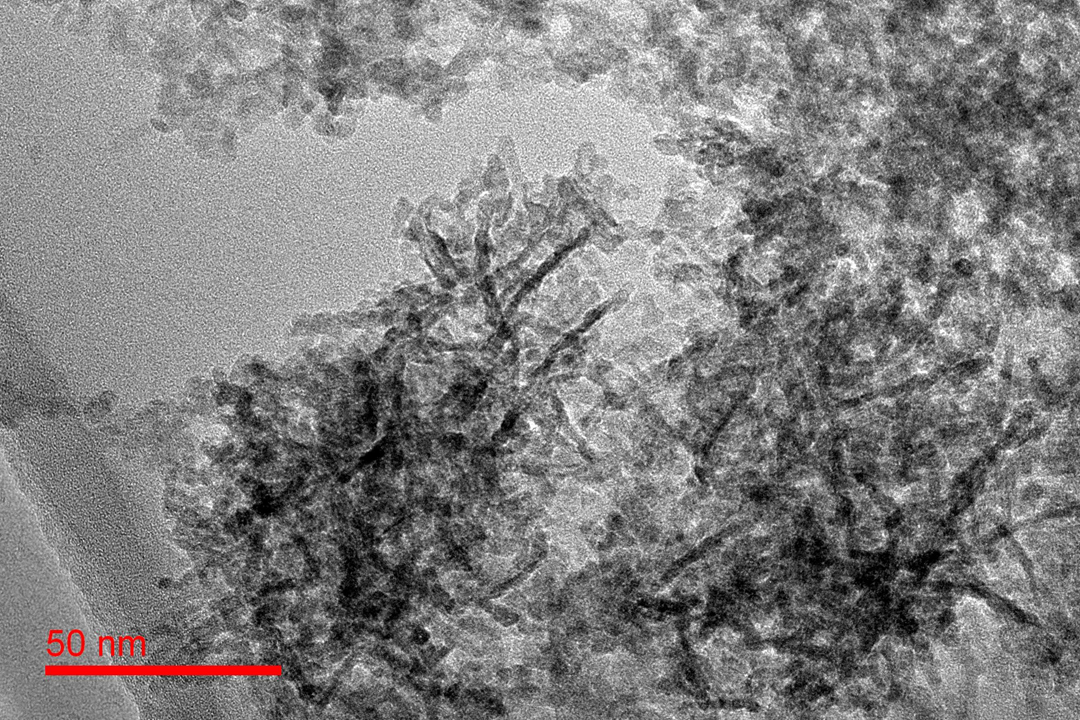


**Fig S11.** TEM image of akaganéite laths with Fh identified in Live FL treatments following two-week incubations.


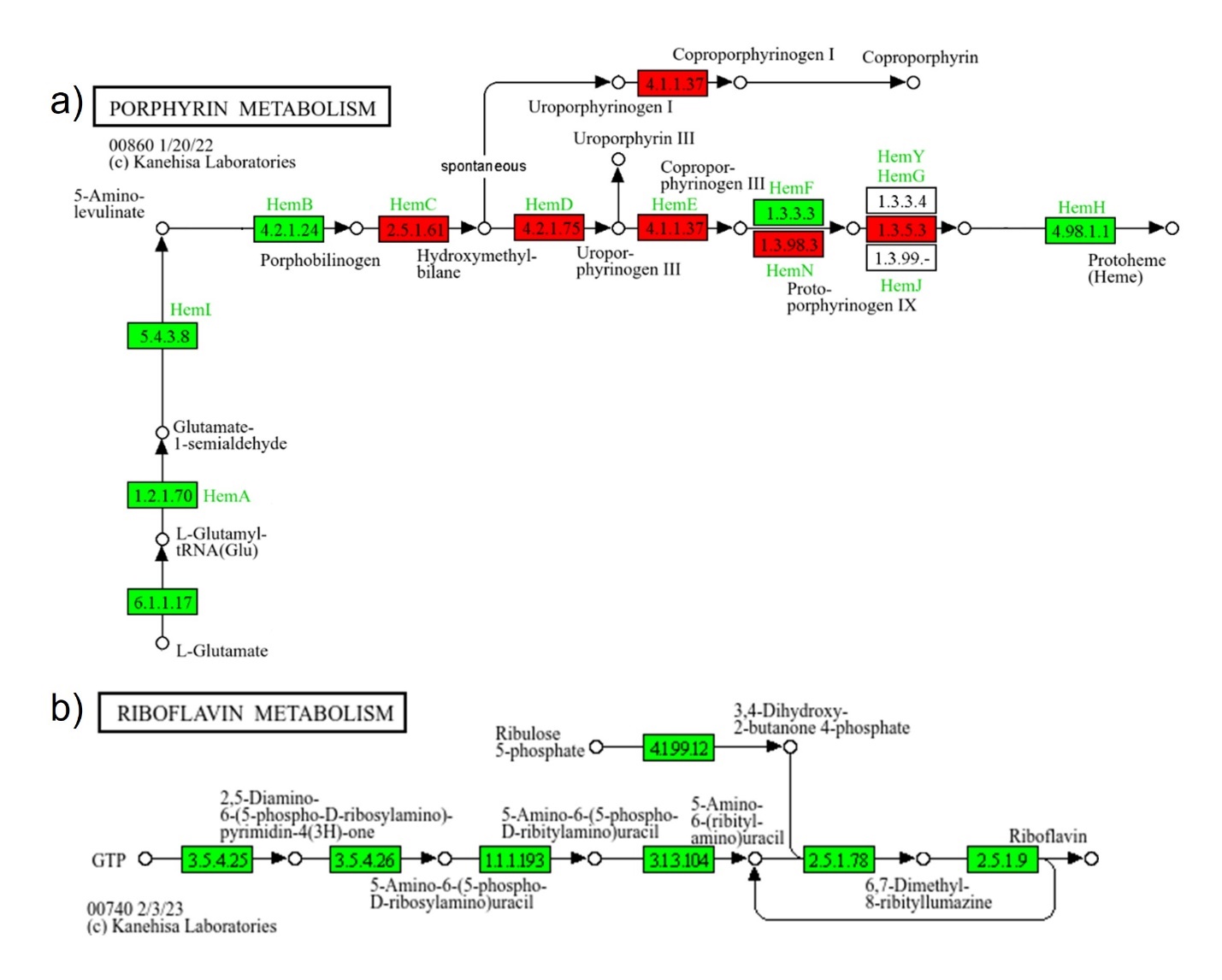


**Fig S12.** Biosynthesis pathways for DIR-relevant compounds. Genes in green are transcribed but are not significantly different between treatments. Genes in red are significantly more transcribed in +Fh treatments. (a) Heme biosynthesis pathway from Porphyrin Metabolism (map00860). (b) Riboflavin biosynthesis pathway from Riboflavin Metabolism (map00740).

**
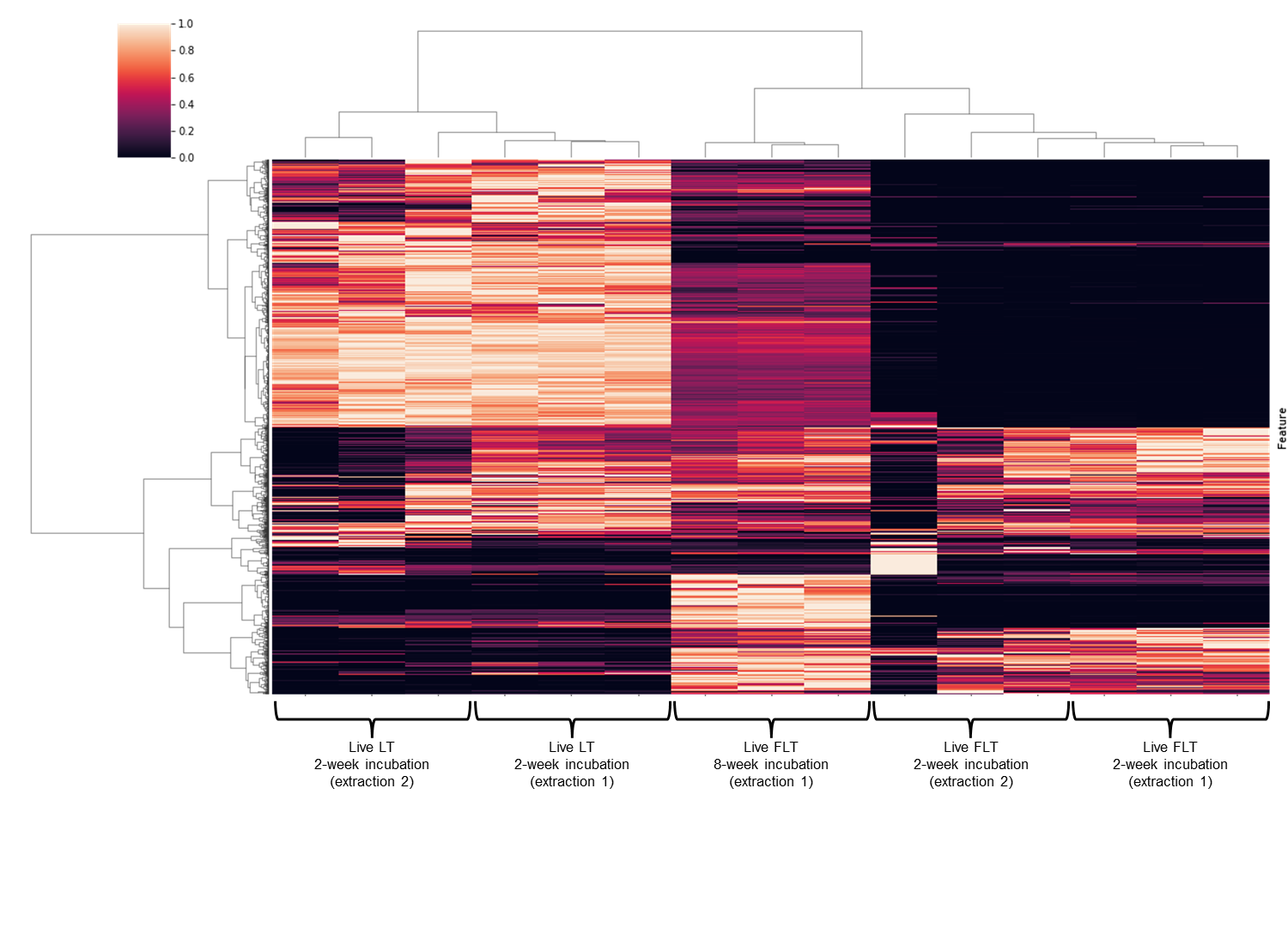
**

**Fig S13.** Hierarchical clustering analysis of normalized molecular feature abundance. Supernatant from LT treatments cluster separately from both two- and eight-week FLT supernatants.

**
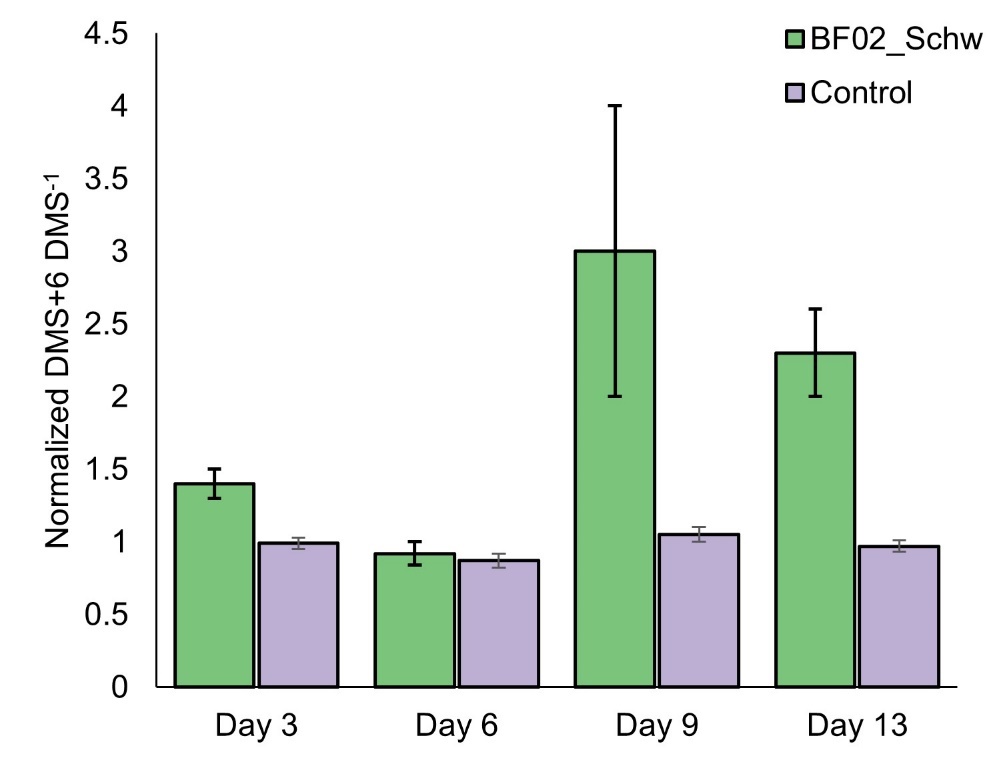
**

**Fig S14.**Reduction of D_6_-DMSO by BF02_Schw and production of D_6_-DMS (M + 6) measured by MIMS.


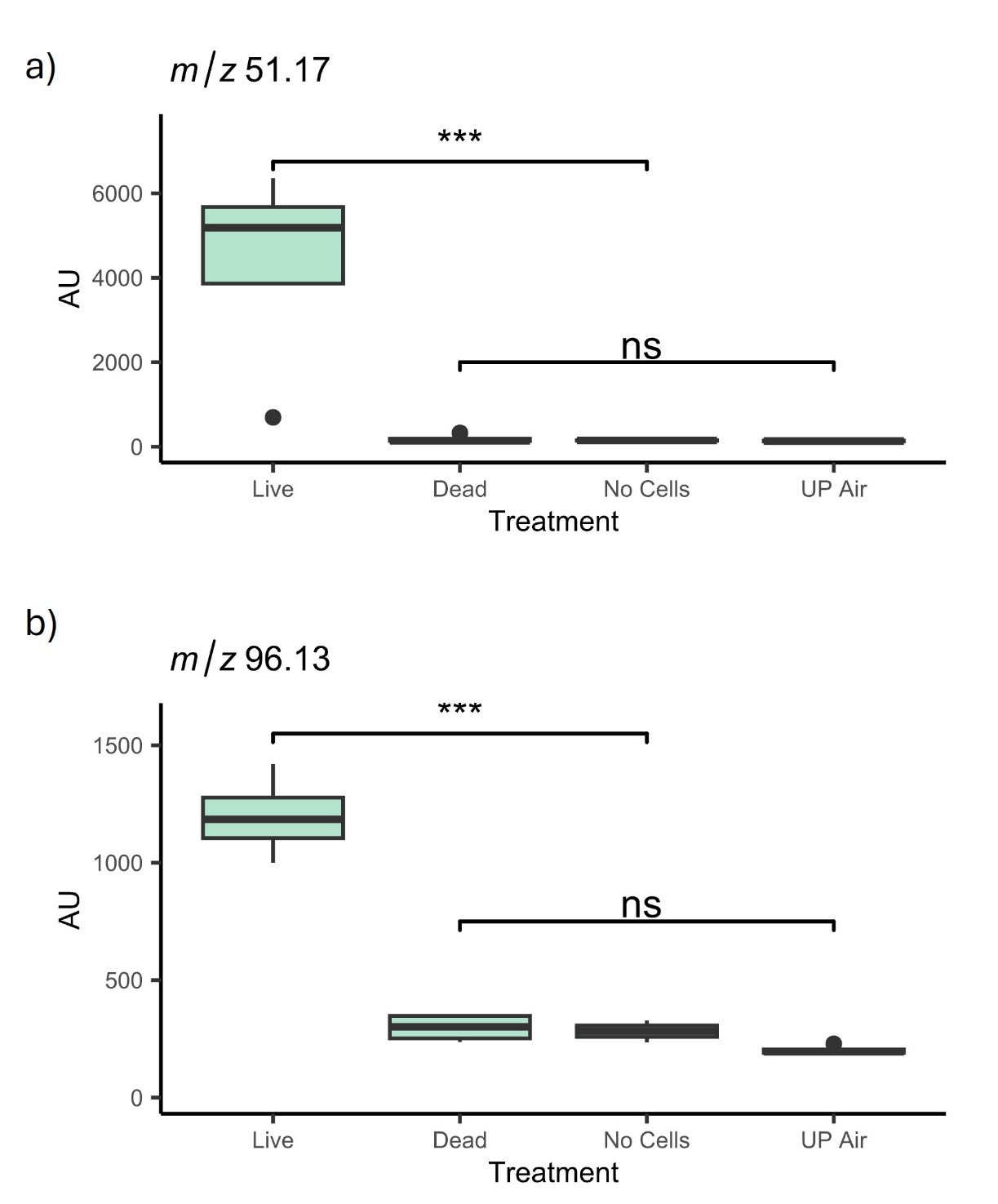


**Fig S15.** Halogenated compounds and putative halogenase produced by BF02_Schw. (a) raw abundance of putative chloromethane and (b) bromomethane features in headspace of aerobic incubations. *ns – not significant*. *UP – ultra pure.*


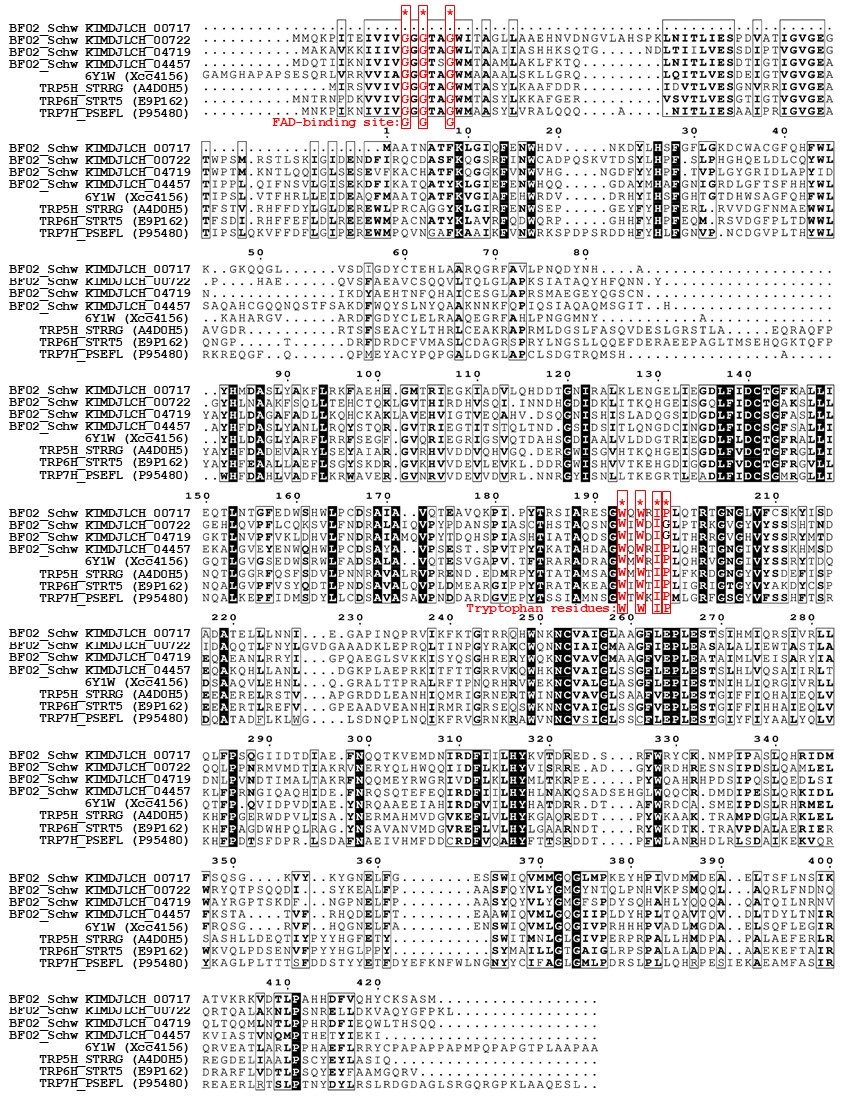


**Fig S16.** Sequence alignment of putative BF02_Schw halogenases against characterized tryptophan halogenases. Conserved FAD-binding site and tryptophan residues are annotated in red. *Xanthomonas campestris* strain B100 putative tryptophan halogenase 6Y1W (Xcc4156) sourced from RCSB PDB. Sequences for *Streptomyces rugosporus* NRRL 21084

tryptophan 5-halogenase TRP5H_STRRG (A4D0H5), *S. toxytricini* NRRL 15443 tryptophan 6-halogenase TRP6H_STRT5 (E9P162), and *Pseudomonas fluorescens* Bl915 tryptophan 7-halogenase TRP7H_PSEFL (P95480) were sourced from UniProt.

**
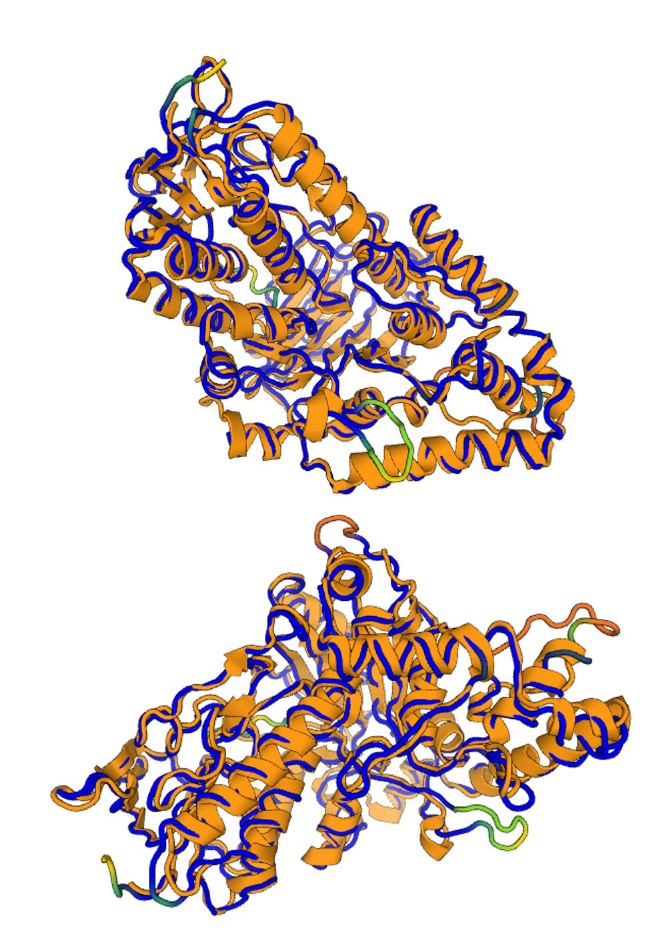
**

**Fig S17.** Predicted structure of BF02_Schw PrnA aligned with flavin-dependent halogenase from *Xanthomonas campestris* (PDB Chain 6Y1W-B; orange). Coloration of PrnA is based on structural conservation, ranging from blue (high) to red (low).

**Table S1.** Mössbauer Parameters: 295K

| 295K | ***CS*** | **QS** | **W** | **Area** | ***CS*** | **QS** | **W** | **Area** | ***CS*** | **QS** | **W** | **Area** |
| --- | --- | --- | --- | --- | --- | --- | --- | --- | --- | --- | --- | --- |
|  | **Fe^3+^** | | | | **Fe^3+^** | | | | **Fe^2+^** | | | |
| FL – No Cells | *0.35* | 0.57 | 0.47 | **67** | *0.35* | 1.02 | 0.46 | **33** |  |  |  |  |
| FL – Dead | *0.35* | 0.56 | 0.41 | **60** | *0.35* | 0.98 | 0.45 | **40** |  |  |  |  |
| FL – Live | *0.35* | 0.55 | 0.55 | **67** | *0.35* | 1.04 | 0.5 | **33** |  |  |  |  |
| FLT – No Cell | *0.35* | 0.55 | 0.45 | **64** | *0.35* | 1 | 0.44 | **36** |  |  |  |  |
| FLT – Dead | *0.35* | 0.58 | 0.45 | **69** | *0.35* | 1.01 | 0.44 | **32** |  |  |  |  |
| FLT – Live | *0.35* | 0.52 | 0.41 | **47** | *0.35* | 0.92 | 0.53 | **48** | *1.31* | 2.02 | 0.58 | **5** |

**Table S2.** Mössbauer Parameters: 4K

| 4K | ***CS*** | **QS** | **W** | Field | **Area** | ***CS*** | **QS** | **W** | Field | **Area** | ***CS*** | **QS** | **W** | Field | **Area** |
| --- | --- | --- | --- | --- | --- | --- | --- | --- | --- | --- | --- | --- | --- | --- | --- |
|  | **Fe^3+^** | | | | | **Fe^3+^** | | | | | **Fe^3+^** | | | | |
| FL – No Cells | *0.44* | -0.06 | 0.58 | 450 | **26** | *0.47* | -0.07 | 0.75 | 480 | **46** | *0.47* | -0.01 | 0.68 | 508 | **28** |
| FL – Dead | *0.44* | -0.1 | 0.75 | 450 | **25** | *0.46* | -0.06 | 0.93 | 482 | **48** | *0.47* | -0.03 | 0.83 | 511 | **27** |
| FL– Live | *0.45* | -0.08 | 0.55 | 463 | **51** | *0.48* | -0.06 | 0.52 | 485 | **25** | *0.47* | -0.02 | 0.55 | 509 | **24** |
| FLT – No Cell | *0.44* | -0.09 | 0.52 | 465 | **42** | *0.48* | -0.09 | 0.67 | 491 | **40** | *0.46* | -0.02 | 0.53 | 514 | **18** |
| FLT – Dead | *0.44* | -0.1 | 0.71 | 456 | **27** | *0.47* | -0.07 | 0.87 | 485 | **44** | *0.47* | -0.04 | 0.78 | 512 | **29** |
| FLT – Live | *0.42* | -0.02 | 0.44 | 476 | **45** | *0.5* | -0.23 | 0.44 | 494 | **22** | *0.45* | -0.05 | 0.52 | 512 | **24** |
| FLT – Live *continued* | **Fe^2.x+^** | | | | | *0.80** | 0.80* | 1.00* | 276* | **6** | *1.30** | 3.00* | 0.90* |  | **3** |

**Table S4.** Differential expression of transcripts of interest.

| Pathway | Gene Name | Gene ID | log2 Fold Change | p_adj_ |
| --- | --- | --- | --- | --- |
| Iron reduction | *omcA* | KIMDJLCH_03019 | 4.79 | < 0.001 |
|  | *mtrC* | KIMDJLCH_03020 | 4.87 | < 0.001 |
|  | *mtrA* | KIMDJLCH_03021 | 5.79 | < 0.001 |
|  | *mtrB* | KIMDJLCH_03022 | 5.02 | < 0.001 |
|  | *cymA* | KIMDJLCH_03378 | 2.81 | 0.005 |
|  | ***cctA*** | **KIMDJLCH_00944** | **2.91** | **0.006 *** |
|  | ***fccA*** | **KIMDJLCH_03490** | **6.29** | **< 0.001 *** |
| Heme biosynthesis | *gltX* | KIMDJLCH_03156 | -0.19 | 1 |
|  | *hemA* | KIMDJLCH_00135 | 0.31 | 1 |
|  | *hemL* | KIMDJLCH_00267 | 0.83 | 1 |
|  | *hemB* | KIMDJLCH_02573 | 1.25 | 1 |
|  | *hemC* | KIMDJLCH_02603 | 2.40 | 0.003 |
|  | *hemD* | KIMDJLCH_02604 | 2.29 | 0.109 |
|  | *hemE* | KIMDJLCH_02557 | 2.77 | 0.001 |
|  | *hemN* | KIMDJLCH_01698 | 3.53 | < 0.001 |
|  | *hemG*_1 | KIMDJLCH_02180 | 3.04 | < 0.001 |
|  | *hemG*_2 | KIMDJLCH_04649 | 3.62 | < 0.001 |
|  | *hemH* | KIMDJLCH_01944 | 1.09 | 1 |
| Thiosulfate reduction | *psrB* | KIMDJLCH_01451 | 8.29 | < 0.001 |
|  | *psrA* | KIMDJLCH_01452 | 7.97 | < 0.001 |
|  | *psrC* | KIMDJLCH_01450 | 7.08 | < 0.001 |
| DMSO reduction | *dmsE* | KIMDJLCH_03495 | 3.58 | < 0.001 |
|  | *dmsF* | KIMDJLCH_03496 | 2.89 | < 0.001 |
|  | *dmsA* | KIMDJLCH_03497 | 3.56 | < 0.001 |
|  | *dmsB* | KIMDJLCH_03498 | 3.64 | < 0.001 |
|  | *dmsG* | KIMDJLCH_03499 | 1.85 | 0.133 |
|  | *dmsH* | KIMDJLCH_03500 | 1.55 | 1 |
| Riboflavin biosynthesis | *ribA* | KIMDJLCH_02823 | -0.24 | 1 |
|  | *ribBA* | KIMDJLCH_00406 | -0.40 | 1 |
|  | *ribD* | KIMDJLCH_00404 | 0.54 | 1 |
|  | *yigB* | KIMDJLCH_02595 | 0.36 | 1 |
|  | *ribC* | KIMDJLCH_01398 | -0.83 | 1 |
|  | *ribE*_1 | KIMDJLCH_00405 | 0.24 | 1 |
|  | *ribE*_2 | KIMDJLCH_00407 | -1.05 | 1 |
| Halogenases | *rebH*_1 | KIMDJLCH_00717 | 0.27 | 1 |
|  | *rebH*_2 | KIMDJLCH_00722 | 0.82 | 1 |
|  | *rebH*_3 | KIMDJLCH_04719 | 1.19 | 1 |
|  | *prnA* | KIMDJLCH_04457 | 1.17 | 1 |
| Housekeeping genes | *gyrA* | KIMDJLCH_01272 | -0.84 | 1 |
|  | *gyrB* | KIMDJLCH_04664 | -0.63 | 1 |
|  | *rpoB* | KIMDJLCH_01798 | 0.50 | 1 |
|  | *rho* | KIMDJLCH_02578 | -0.12 | 1 |

*Log2FoldChange is relative to LT (i.e., positive values are higher in FLT treatments).*

**Table S5.** Abundance and putative identities of VOC features differentially abundant between treatments.

**Day 14**

| ***m/z*** | **Log(2) Fold Change** | **ANOVA (*p*)** | **Average Normalized Peak Height** | | | | | | | | **Putative ID's (assigned by Progenesis MALDI)** |
| --- | --- | --- | --- | --- | --- | --- | --- | --- | --- | --- | --- |
|  |  |  | **Blank** | **UHP N_2_** | **FLT,**  **No Cell** | **FLT,**  **Dead** | **FLT,**  **Live** | **LT,**  **No Cell** | **LT,**  **Dead** | **LT,**  **Live** |  |
| 41.03 | 128.78 | 1.47E-06 | 0.118 | 2.247 | 8.924 | 6.364 | 7.896 | 7.588 | 4.549 | 15.181 | Cyclopropene (C_3_H_4_) Propyne (C_3_H_4_) Propa-1,2-diene (C_3_H_4_) |
| 42.43 | 64853.36 | 0.000321 | 0.016 | 0.03 | 0.1 | 0.077 | 0.215 | 2.551 | 12.954 | 1024.237 | Acetonitrile (CH_3_CN) |
| 45.26 | 169.45 | <1.1E-16 | 2.131 | 60.751 | 237.229 | 166.065 | 173.916 | 342.092 | 309.901 | 361.147 | Ethanal (CH_3_CHO) Ethylene oxide (C_2_H_4_O) Propane (CH_3_CH_2_CH_3_) |
| 53.72 | 19.23 | 3.17E-12 | 0.231 | 1.215 | 4.45 | 3.759 | 3.835 | 2.951 | 2.282 | 0.953 | 1-Oxoprop-2-ynyl (C_3_HO) 2-Propenenitrile (C_3_H_3_N) |
| 55.09 | 156.58 | 5.01E-09 | 0.043 | 0.803 | 2.052 | 1.601 | 4.912 | 1.78 | 1.274 | 6.775 | 1,3-Butadiene (C_4_H_6_) 1,2-Butadiene (C_4_H_6_) Cyclobutene (C_4_H_6_) 1-Butyne (C_4_H_6_) 2-Butyne (C_4_H_6_) 1-Methylcyclopropene (C_4_H_6_) |
| 56.58 | 372.91 | 5.08E-12 | 0.208 | 2.39 | 32.63 | 45.62 | 21.15 | 61.44 | 77.541 | 21.266 | 1-Oxoprop-2-enyl (C_3_H_3_O)  Propanenitrile ( C_3_H_5_N)  Vinylimine (C_3_H_5_N)  Propargylamine (C_3_H_5_N)  Cyclopropanimine (C_3_H_5_N) |
| 59.4 | 89.86 | 1.63E-12 | 0.084 | 2.922 | 7.554 | 6.422 | 6.939 | 2.951 | 2.044 | 2.092 | Acetone (CH_3_COCH_3_) Ethanedial (C_2_H_2_O_2_) Propanal (CH_3_CH_2_CHO) Propen-2-ol (CH_3_COHCH_2_) 2-Propen-1-ol (CH_2_CHCH_2_OH) Dimethyldiazene (C_2_H_6_N_2_) Butane (C_4_H_10_) Methylpropane (C_4_H_10_) |
| 62.25 | 2290.81 | 0.000421 | 0.007 | 0.036 | 0.465 | 0.424 | 3.916 | 0.087 | 0.171 | 16.97 | N-Methoxy-methanamine (C_2_H_7_NO) Monoethanolamine (C_2_H_7_NO) N-Hydroxy-N-methylmethanamine (C_2_H_7_NO) Thioformamide (CH_3_NS) |
| 76.69 | 47.08 | 6.99E-14 | 0.046 | 1.498 | 1.85 | 1.558 | 2.145 | 1.194 | 1.1 | 1.459 | Acetohydroxamic acid (C_2_H_5_NO_2_) Glycine (C_2_H_5_NO_2_) Glycoamide (C_2_H_5_NO_2_) Methylcarbamate (C_2_H_5_NO_2_) Hydrazinecarboxamide (CH_5_N_3_O) 2-Methoxyethanamine (C_3_H_9_NO) Trimethylamine N-oxide (C_3_H_9_NO) Dimethylaminomethanol (C_3_H_9_NO) 3-Amino-1-propanol (C_3_H_9_NO) 2-Amino-1-propanol (C_3_H_9_NO) 1-Amino-2-propanol (C_3_H_9_NO) Ethanethioamide (C_2_H_5_NS) |
| 79.55 | 199.51 | 1.09E-06 | 0.025 | 0.946 | 5.041 | 3.692 | 4.462 | 1.832 | 0.798 | 0.749 | Benzene (C_6_H_6_) Dimethylsulfoxide (CH_3_SOCH_3_) 2-Mercaptoethanol (CH_2_OHCH_2_SH) |
| 82.44 | 77.05 | 3.10E-05 | 0.009 | 0.093 | 0.392 | 0.306 | 0.708 | 0.377 | 0.354 | 0.468 | 1-Methyl-(1H)-pyrrole (C_5_H_7_N) 2-Methyl-(1H)-pyrrole (C_5_H_7_N) 3-Methyl-(1H)-pyrrole (C_5_H_7_N) Methallyl cyanide (C_5_H_7_N) |
| 94.12 | 17.85 | 0.000226 | 0.026 | 0.075 | 0.461 | 0.301 | 0.401 | 0.171 | 0.072 | 0.056 | 3-Methyl-pyridine (C_5_H_6_N_2_) 3-Methyl-pyridine (C_5_H_6_N_2_) Phenylamine (C_6_H_7_N) |
| 99.96 | 127.71 | 0.000911 | 0.01 | 0.7 | 1.315 | 0.852 | 1.004 | 0.35 | 0.192 | 0.12 | Piperidinone (C_5_H_9_NO) 1-Butylisocyanate (C_5_H_9_NO) Isothiocyanatocyclopropane (C_4_H_5_NS) Allylisothiocyanate (C_4_H_5_NS) 5-Methyl-isothiozole (C_4_H_5_NS) |
| 111.56 | 431.53 | 2.33E-08 | 0.036 | 0.452 | 0.467 | 7.108 | 0.762 | 0.561 | 15.401 | 0.201 | 1-(2-Furany)-ethanone (C_6_H_6_O_2_) 2-Methyl-2-furancarboxaldehyde (C_6_H_6_O_2_) 1-(1H-Pyrazol-4-yl)-ethanone (C_5_H_6_N_2_O) 2-Ethyl-5-methylfuran (C_7_H_10_O) 2,3,5-Trimethylfuran (C_7_H_10_O) 3-Methyl-2-cyclohexen-1-one (C_7_H_10_O) |
| 114.49 | 1205.38 | 3.20E-08 | 0.008 | 0.167 | 0.283 | 4.58 | 0.472 | 0.3 | 9.822 | 0.041 | 1-Methyl-2-piperidinone (C_6_H_11_NO) 4-Isothiocyanato-1-butene (C_5_H_7_NS) |
| 117.44 | 238.07 | 1.28E-07 | 0.006 | 0.194 | 0.23 | 0.809 | 0.18 | 0.149 | 1.545 | 0.062 | Nitrosomorpholine (C_4_H_8_H_2_O_2_) Hexanoic acid (C_6_H_12_O_2_) 5-Methyl-3-hexanol (C_7_H_16_O) |
| 120.58 | 89.46 | 0.00015 | 0.008 | 0.119 | 0.744 | 0.415 | 0.446 | 0.12 | 0.048 | 0.051 | Trichoromethane (CHC_3_) |
| 123.35 | 34.44 | 2.22E-16 | 0.017 | 0.491 | 0.521 | 0.499 | 0.507 | 0.578 | 0.547 | 0.473 | Benzoic acid (C_7_H_6_O_2_) 2-Ethyl-phenol (C_8_H_8_O) 4-Ethyl-phenol (C_8_H_8_O) |
| 141.33 | 52.51 | 3.60E-05 | 0.006 | 0.131 | 0.331 | 0.212 | 0.214 | 0.046 | 0.033 | 0.033 | 2,2,6-Trimethylcyclohexanone (C_9_H_16_O) |
| 190.55 | 10.51 | 3.23E-08 | 0.056 | 0.28 | 0.505 | 0.479 | 0.587 | 0.109 | 0.128 | 0.134 | N-Acetyl-L-glutamic acid (C_7_H_11_NO_5_) a-Cyano-3-hydroxycinnamic acid (C_10_H_7_NO_3_) 1H-Indole-3-acetic acid methyl ester (C_11_H_11_NO_2_) 1H-Indole-3-propanoic acid (C_11_H_11_NO_2_) Tricyclazole (C_9_H_7_N_3_S) Lesquerellin (C_8_H_15_NS_2_) |
|  |  |  |  |  |  |  |  |  |  |  |  |

**Day 56**

| ***m/z*** | **Log(2) Fold Change** | **ANOVA (*p*)** | **Average Normalized Peak Height** | | | | | | | | **Putative ID's (assigned by Progenesis MALDI)** |
| --- | --- | --- | --- | --- | --- | --- | --- | --- | --- | --- | --- |
|  |  |  | **Blank** | **UHP N_2_** | **FLT,**  **No Cell** | **FLT,**  **Dead** | **FLT,**  **Live** | **LT,**  **No Cell** | **LT,**  **Dead** | **LT,**  **Live** |  |
| 36.91 | 23.75 | 8.88E-16 | 0.057 | 0.286 | 0.208 | 0.199 | 1.345 | 0.174 | 0.175 | 0.865 |  |
| 46.68 | 3.1 | 2.20E-09 | 0.095 | 0.139 | 0.179 | 0.17 | 0.295 | 0.143 | 0.125 | 0.151 | Nitrosomethane (CH_3_NO) Dimethylamine (CH_3_NHCH_3_) Ethylamine (CH_3_CH_2_NH_2_) |
| 48.09 | 5.91 | <1.1E-16 | 0.501 | 1.566 | 1.602 | 1.557 | 2.961 | 1.52 | 1.444 | 1.724 | N-Methyl-hydroxylamine (CH_5_NO) O-Methyl-hydroxylamine (CH_5_NO) |
| 55.11 | 148.97 | <1.1E-16 | 0.016 | 0.231 | 0.09 | 0.091 | 0.672 | 0.098 | 0.14 | 2.334 | 1,3-Butadiene (C_4_H_6_) 1,2-Butadiene (C_4_H_6_) Cyclobutene (C_4_H_6_) 1-Butyne (C_4_H_6_) 2-Butyne (C_4_H_6_) 1-Methylcyclopropene (C_4_H_6_) |
| 56.55 | 433.61 | <1.1E-16 | 0.166 | 1.233 | 5.006 | 4.932 | 27.768 | 7.149 | 6.575 | 72.12 | 1-Oxoprop-2-enyl (C_3_H_3_O)  Propanenitrile ( C_3_H_5_N)  Vinylimine (C_3_H_5_N)  Propargylamine (C_3_H_5_N)  Cyclopropanimine (C_3_H_5_N) |
| 59.39 | 183.57 | <1.1E-16 | 0.025 | 0.111 | 0.128 | 0.115 | 4.612 | 0.08 | 0.097 | 0.75 | Acetone (CH_3_COCH_3_) Ethanedial (C_2_H_2_O_2_) Propanal (CH_3_CH_2_CHO) Propen-2-ol (CH_3_COHCH_2_) 2-Propen-1-ol (CH_2_CHCH_2_OH) Dimethyldiazene (C_2_H_6_N_2_) Butane (C_4_H_10_) Methylpropane (C_4_H_10_) |
| 60.82 | 78.05 | 1.29E-09 | 0.003 | 0.005 | 0.005 | 0.012 | 0.228 | 0.004 | 0.015 | 0.08 |  |
| 62.27 | 1345.47 | <1.1E-16 | 0.004 | 0.013 | 0.015 | 0.024 | 5.533 | 0.012 | 0.015 | 0.625 | N-Methoxy-methanamine (C_2_H_7_NO) Monoethanolamine (C_2_H_7_NO) N-Hydroxy-N-methylmethanamine (C_2_H_7_NO) Thioformamide (CH_3_NS) |
| 63.71 | 9.08 | 4.44E-16 | 0.015 | 0.014 | 0.019 | 0.02 | 0.125 | 0.016 | 0.018 | 0.029 | 1,2-Ethanediol (C_2_H_6_O_2_) Dimethylsulfide (C_2_H_6_S) Ethanethiol (C_2_H_6_S) |
| 65.13 | 31.58 | 1.11E-16 | 0.008 | 0.014 | 0.017 | 0.016 | 0.267 | 0.014 | 0.012 | 0.041 |  |
| 72.22 | 1.31 | 1.19E-06 | 0.928 | 1.069 | 1.206 | 1.208 | 1.217 | 1.007 | 0.999 | 1.016 | 2-Hydroxy-propanenitrile (C_3_H_5_NO) 3-Hydroxy-propanenitrile (C_3_H_5_NO) Isocyanatoethane (C_3_H_5_NO) Methoxyacetonitrile (C_3_H_5_NO) 2-Propanamide (C_3_H_5_NO) Pyrrolidine (C_4_H_9_N) 2-Methyl-2-propen-1-amine (C_4_H_9_N) N-Methyl-2-propen-1-amine (C_4_H_9_N) N-Methylallylamine (C_4_H_9_N) |
| 76.72 | 95.9 | <1.1E-16 | 0.026 | 0.243 | 0.143 | 0.137 | 2.455 | 0.143 | 0.163 | 2.023 | Acetohydroxamic acid (C_2_H_5_NO_2_) Glycine (C_2_H_5_NO_2_) Glycoamide (C_2_H_5_NO_2_) Methylcarbamate (C_2_H_5_NO_2_) Hydrazinecarboxamide (CH_5_N_3_O) 2-Methoxyethanamine (C_3_H_9_NO) Trimethylamine N-oxide (C_3_H_9_NO) Dimethylaminomethanol (C_3_H_9_NO) 3-Amino-1-propanol (C_3_H_9_NO) 2-Amino-1-propanol (C_3_H_9_NO) 1-Amino-2-propanol (C_3_H_9_NO) Ethanethioamide (C_2_H_5_NS) |
| 78.17 | 12.3 | 2.01E-11 | 0.009 | 0.021 | 0.02 | 0.019 | 0.106 | 0.017 | 0.017 | 0.088 |  |
| 83.82 | 30.84 | <1.1E-16 | 0.004 | 0.01 | 0.006 | 0.007 | 0.131 | 0.008 | 0.008 | 0.061 |  |
| 105.76 | 18.54 | <1.1E-16 | 0.008 | 0.02 | 0.03 | 0.027 | 0.152 | 0.03 | 0.029 | 0.081 | Diethanolamine (C_4_H_11_NS) 2-Amino-1,3-butanediol (C_4_H_11_NS) N-(Methyl)mercaptoacetamide (C_3_H_7_NOS) DL-a-aminothiopropionic acid (C_3_H_7_NOS) b-Mercaptopropionamide (C_3_H_7_NOS) 2-(Dimethylamino)-ethanethiol (C_4_H_11_NS) |
| 111.61 | 210.58 | <1.1E-16 | 0.009 | 0.323 | 0.062 | 1.359 | 0.096 | 0.056 | 1.91 | 0.171 | 1-(2-Furany)-ethanone (C_6_H_6_O_2_) 2-Methyl-2-furancarboxaldehyde (C_6_H_6_O_2_) 1-(1H-Pyrazol-4-yl)-ethanone (C_5_H_6_N_2_O) 2-Ethyl-5-methylfuran (C_7_H_10_O) 2,3,5-Trimethylfuran (C_7_H_10_O) 3-Methyl-2-cyclohexen-1-one (C_7_H_10_O) |
| 114.55 | 338.54 | <1.1E-16 | 0.004 | 0.028 | 0.008 | 0.89 | 0.012 | 0.006 | 1.208 | 0.011 | 1-Methyl-2-piperidinone (C_6_H_11_NO) 4-Isothiocyanato-1-butene (C_5_H_7_NS) |
| 117.49 | 75.17 | 1.11E-16 | 0.003 | 0.01 | 0.006 | 0.145 | 0.005 | 0.005 | 0.198 | 0.006 | Nitrosomorpholine (C_4_H_8_H_2_O_2_) Hexanoic acid (C_6_H_12_O_2_) 5-Methyl-3-hexanol (C_7_H_16_O) |

**Table S6.** Protein Data Bank (PDB) matches with structural similarity to predicted BF02_Schw PrnA (KIMDJLCH_04457) structure.

| Protein description | Chain | Z score | Root mean square deviation | Lali (# of equivalent residues) | # of residues in target structure | % Identity to PDB match |
| --- | --- | --- | --- | --- | --- | --- |
| Putative Tryptophan Halogenase | 6y1w-b | 52.6 | 1.1 | 479 | 493 | 48 |
| Putative Tryptophan Halogenase | 6y1w-a | 51.8 | 1.2 | 483 | 505 | 48 |
| Tryptophan Halogenase Superfamily | 6frl-a | 50.5 | 1.4 | 483 | 491 | 46 |
| Tryptophan Halogenase Superfamily | 6frl-b | 50.2 | 1.4 | 481 | 492 | 46 |
| Tryptophan 5-Halogenase | 2wet-c | 47.9 | 1.7 | 476 | 494 | 33 |
| Tryptophan 5-Halogenase | 2wet-d | 47.9 | 1.7 | 476 | 494 | 33 |
| Tryptophan 5-Halogenase | 2wet-a | 47.8 | 1.6 | 476 | 496 | 33 |
| Tryptophan 5-Halogenase | 8fov-a | 47.7 | 1.8 | 480 | 496 | 33 |
| Tryptophan 5-Halogenase | 8fov-b | 47.6 | 1.7 | 482 | 500 | 33 |
| Tryptophan 6-Halogenase | 5hy5-b | 47.4 | 1.8 | 473 | 490 | 33 |
| Tryptophan 5-Halogenase | 2wes-c | 47.0 | 1.7 | 476 | 496 | 33 |
| Tryptophan 5-Halogenase | 2wes-b | 47.0 | 1.7 | 476 | 496 | 33 |
| Tryptophan 5-Halogenase | 2wes-d | 47.0 | 1.7 | 476 | 496 | 33 |
| Tryptophan 5-Halogenase | 2wes-a | 47.0 | 1.7 | 476 | 496 | 33 |
| Tryptophan 5-Halogenase | 2wet-b | 46.8 | 1.7 | 474 | 502 | 33 |
| Tryptophan Halogenase | 6nsd-b | 46.6 | 1.9 | 474 | 498 | 33 |
| Tryptophan Halogenase | 6nsd-a | 46.4 | 1.9 | 475 | 503 | 33 |
| Flavin-Dependent Tryptophan Halogenase PrnA | 4z43-a | 46.2 | 1.9 | 481 | 505 | 34 |
| Tryptophan 5-Halogenase | 8fox-d | 46.0 | 1.9 | 477 | 491 | 33 |
| Tryptophan Halogenase PrnA | 2ard-a | 45.9 | 1.9 | 481 | 503 | 34 |
| Tryptophan 5-Halogenase | 8fox-a | 45.9 | 1.8 | 470 | 488 | 33 |
| Tryptophan 5-Halogenase | 8fox-c | 45.9 | 2 | 476 | 489 | 34 |
| Tryptophan 5-Halogenase | 8fox-b | 45.7 | 1.9 | 474 | 495 | 33 |
| Tryptophan 6-Halogenase | 5hy5-a | 45.6 | 1.9 | 476 | 510 | 33 |
| Tryptophan 6-Halogenase | 6sls-b | 45.6 | 1.8 | 477 | 517 | 31 |
| Tryptophan Halogenase PrnA | 2jkc-a | 45.4 | 1.9 | 482 | 517 | 34 |
| Tryptophan 5-Halogenase | 2weu-c | 45.1 | 1.9 | 474 | 500 | 32 |
| Tryptophan 5-Halogenase | 2weu-a | 45.1 | 1.7 | 467 | 495 | 33 |
| Tryptophan Halogenase PrnA | 2apg-a | 45.1 | 1.9 | 479 | 517 | 34 |
| Tryptophan 6-Halogenase | 6sls-a | 45.0 | 1.8 | 479 | 524 | 30 |
| Tryptophan Halogenase PrnA | 2aqj-a | 45.0 | 1.9 | 481 | 518 | 34 |
| Tryptophan 5-Halogenase | 2weu-d | 45.0 | 1.7 | 469 | 499 | 32 |
| Tryptophan Halogenase PrnA | 2ar8-a | 45.0 | 1.9 | 479 | 518 | 34 |
| Tryptophan 6-Halogenase | 7cu2-b | 44.9 | 1.9 | 480 | 520 | 31 |
| Flavin-Dependent Tryptophan Halogenase PrnA | 4z44-a | 44.9 | 2 | 481 | 516 | 34 |
| Tryptophan 6-Halogenase | 6slt-b | 44.7 | 1.8 | 477 | 525 | 31 |
| Tryptophan 5-Halogenase | 2weu-b | 44.7 | 1.8 | 471 | 500 | 32 |
| Thermophilic Tryptophan Halogenase | 5lv9-b | 44.3 | 1.9 | 474 | 513 | 34 |
| Thermophilic Tryptophan Halogenase | 5lv9-a | 44.2 | 1.9 | 467 | 509 | 34 |
| Tryptophan 6-Halogenase | 7cu1-a | 44.1 | 1.8 | 476 | 535 | 30 |
| Tryptophan 6-Halogenase ThaL | 6h43-b | 43.6 | 1.9 | 477 | 521 | 31 |
| Flavin-Dependent Tryptophan Halogenase RebH | 6p00-b | 43.5 | 1.9 | 476 | 519 | 32 |
| Tryptophan 6-Halogenase | 7aqv-a | 43.5 | 1.9 | 473 | 520 | 31 |
| Flavin-Dependent Tryptophan Halogenase RebH | 6p00-a | 43.5 | 1.9 | 474 | 518 | 31 |
| Tryptophan 6-Halogenase | 8ad8-b | 43.5 | 1.9 | 473 | 515 | 30 |
| Tryptophan 6-Halogenase | 7cu1-b | 43.5 | 1.9 | 477 | 524 | 30 |
| Tryptophan 6-Halogenase | 7cu2-a | 43.4 | 2 | 478 | 522 | 31 |
| Flavin-Dependent Tryptophan Halogenase RebH | 6p2v-b | 43.4 | 1.8 | 474 | 521 | 32 |
| Tryptophan 6-Halogenase | 8ttk-d | 43.3 | 1.9 | 474 | 517 | 32 |
| Tryptophan 6-Halogenase | 8ttk-b | 43.2 | 1.8 | 472 | 519 | 32 |
| Tryptophan Halogenase | 2oal-b | 43.2 | 1.9 | 477 | 528 | 32 |
| Tryptophan 6-Halogenase | 8tti-d | 43.2 | 1.8 | 470 | 519 | 32 |
| Tryptophan 6-Halogenase | 8ttk-c | 43.2 | 1.9 | 474 | 519 | 32 |
| Tryptophan 6-Halogenase | 6ib5-b | 43.2 | 1.9 | 476 | 524 | 31 |
| Flavin-Dependent Tryptophan Halogenase RebH | 6p2v-a | 43.2 | 1.9 | 476 | 523 | 31 |
| Tryptophan Halogenase | 2oam-b | 43.2 | 1.9 | 476 | 524 | 32 |
| Tryptophan 6-Halogenase | 8ad8-a | 43.2 | 2 | 478 | 524 | 29 |
| Tryptophan 6-Halogenase | 7aqu-b | 43.2 | 1.9 | 477 | 527 | 31 |
| Tryptophan 6-Halogenase | 8ttk-a | 43.1 | 1.8 | 472 | 521 | 32 |
| Tryptophan 6-Halogenase | 6h44-b | 43.1 | 1.9 | 478 | 529 | 31 |
| Tryptophan Halogenase | 2o9z-a | 43.1 | 1.9 | 474 | 524 | 31 |
| Flavin-Dependent Tryptophan Halogenase RebH | 7ju0-b | 43.1 | 1.9 | 476 | 528 | 31 |
| Tryptophan 6-Halogenase | 8ad7-b | 43.1 | 1.9 | 475 | 525 | 31 |
| Tryptophan 6-Halogenase | 8tti-c | 43.1 | 1.8 | 471 | 520 | 32 |
| Tryptophan Halogenase | 2oa1-a | 43.0 | 1.9 | 476 | 529 | 32 |
| Tryptophan Halogenase | 2oam-a | 43.0 | 1.9 | 475 | 523 | 31 |
| Tryptophan 6-Halogenase | 6ib5-a | 43.0 | 2 | 477 | 526 | 30 |
| Tryptophan 6-Halogenase | 7aqv-b | 43.0 | 2 | 477 | 530 | 30 |
| Flavin-Dependent Tryptophan Halogenase RebH | 7ju0-a | 42.9 | 1.9 | 476 | 527 | 31 |
| Tryptophan 6-Halogenase | 8tti-a | 42.9 | 1.8 | 472 | 522 | 32 |
| Tryptophan Halogenase | 2oa1-b | 42.9 | 2 | 476 | 527 | 32 |
| Tryptophan Halogenase | 2e4g-b | 42.9 | 1.9 | 477 | 529 | 32 |
| Tryptophan Halogenase | 2o9z-b | 42.8 | 2 | 475 | 526 | 31 |
| Tryptophan 6-Halogenase | 8ttj-d | 42.8 | 1.9 | 472 | 521 | 32 |
| Tryptophan 6-Halogenase | 6h44-a | 42.8 | 2 | 476 | 529 | 30 |
| Tryptophan 6-Halogenase ThaL | 6h43-a | 42.8 | 1.9 | 476 | 528 | 31 |
| Tryptophan 6-Halogenase | 6ul2-a | 42.8 | 1.9 | 477 | 527 | 32 |
| Tryptophan Halogenase | 2oal-a | 42.8 | 2 | 476 | 527 | 31 |
| Flavin-Dependent Tryptophan Halogenase RebH | 4lu6-b | 42.8 | 1.9 | 476 | 527 | 32 |
| Tryptophan 6-Halogenase | 8ttj-c | 42.8 | 1.9 | 473 | 522 | 32 |
| Tryptophan Halogenase | 2pyx-b | 42.7 | 2.4 | 476 | 525 | 32 |
| Tryptophan Halogenase | 2e4g-a | 42.7 | 2 | 477 | 529 | 31 |
| Tryptophan 6-Halogenase | 8ttj-a | 42.7 | 1.9 | 472 | 523 | 32 |
| Tryptophan 6-Halogenase | 6ul2-d | 42.7 | 1.8 | 468 | 517 | 32 |
| Tryptophan 6-Halogenase | 8ttj-b | 42.6 | 1.9 | 472 | 523 | 32 |
| Tryptophan Halogenase | 2pyx-a | 42.6 | 2.4 | 476 | 526 | 32 |
| Flavin-Dependent Tryptophan Halogenase RebH | 4lu6-a | 42.6 | 2 | 476 | 527 | 32 |
| Tryptophan 6-Halogenase | 6ul2-b | 42.6 | 1.8 | 471 | 521 | 32 |
| Tryptophan 6-Halogenase | 7aqu-a | 42.6 | 2 | 476 | 530 | 31 |
| Tryptophan 6-Halogenase | 8tti-b | 42.6 | 1.8 | 472 | 522 | 32 |
| Tryptophan 6-Halogenase | 7cu0-b | 42.6 | 2 | 475 | 531 | 30 |
| Tryptophan 6-Halogenase | 8ad7-a | 42.6 | 2 | 477 | 529 | 30 |
| Tryptophan 6-Halogenase | 7cu0-a | 42.5 | 2 | 475 | 532 | 30 |
| Tryptophan 6-Halogenase | 6slt-a | 42.5 | 2 | 477 | 529 | 30 |
| Tryptophan 6-Halogenase | 6ul2-c | 42.5 | 1.9 | 471 | 520 | 32 |
| Tryptophane-5-Halogenase | 5uao-c | 40.7 | 2.1 | 459 | 514 | 25 |
| Tryptophane-5-Halogenase | 5uao-d | 40.6 | 2.2 | 461 | 514 | 25 |
| Tryptophane-5-Halogenase | 5uao-a | 40.3 | 2.2 | 460 | 519 | 25 |
| VirX1 | 6qgm-c | 40.2 | 2.3 | 474 | 522 | 28 |
| Tryptophane-5-Halogenase | 5uao-b | 40.2 | 2.2 | 459 | 516 | 25 |
